# Efficacy and safety of treatments in HR+/HER2- advanced breast cancer after CDK4/6 inhibitor progression: a network meta-analysis and scoping review

**DOI:** 10.1186/s12885-026-15632-z

**Published:** 2026-02-07

**Authors:** Li Zhang, Fan Sun, Xiaorui Wang, Weipeng Zhao, Yongsheng Jia, Haotian Wang, Yuqi Han, Zhongsheng Tong

**Affiliations:** Department of Breast Oncology, Tianjin Medical University Cancer Institute & Hospital, Huanhuxi Road, Hexi District, Tianjin, 300060 China

**Keywords:** HR+/HER2- advanced breast cancer, Post CDK4/6 inhibitors, Scoping review, Network Meta-Analysis

## Abstract

**Background:**

Considering limited therapeutic options and lack of consensus following cyclin-dependent kinase 4/6 inhibitor (CDK4/6i) progression, we evaluated efficacy and safety of available treatments for hormone receptor-positive (HR+)/HER2- advanced breast cancer patients who progressed after CDK4/6i therapy.

**Methods:**

PubMed, Embase, and the Cochrane Library were searched for relevant studies published between 2015 and 2024. Randomized controlled trials (RCTs) were included. Eligible treatments included endocrine therapy (ET) with targeted agents, CDK4/6i, oral selective estrogen receptor degraders (SERDs), and antibody–drug conjugates (ADCs), ET monotherapy, and chemotherapy. Bayesian hierarchical models were applied using a fixed-effects model. Descriptive statistical methods were used to summarize the effect of each treatment. The primary outcome was progression-free survival (PFS), while secondary outcomes were objective response rate (ORR) and adverse events (AEs).

**Results:**

A total of 16 RCTs (*n* = 5,076) were included. Due to the absence of closed loops, two small networks formed based on ET monotherapy and chemotherapy. The camizestrant (HR = 0.49, 95% CI: 0.32–0.76), ribociclib plus ET (HR = 0.57, 95% CI: 0.39–0.84), capivasertib plus ET (HR = 0.59, 95% CI: 0.48–0.72), elacestrant (HR = 0.70, 95% CI: 0.55–0.89), and abemaciclib plus ET (HR = 0.73, 95% CI: 0.57–0.94) showed superior PFS compared to ET monotherapy. Camizestrant, ribociclib plus ET, and capivasertib plus ET demonstrated a significantly better PFS compared with amcenestrant or palbociclib plus ET. ADCs including trastuzumab deruxtecan (T-DXd) (PFS HR = 0.59, ORR OR = 3.53), sacituzumab govitecan (SG) (PFS HR = 0.63, ORR OR = 1.63), and datopotamab deruxtecan (Dato-DXd) (PFS HR = 0.63, ORR OR = 1.94) demonstrated better efficacy in both PFS and ORR compared with chemotherapy. Descriptive statistical analysis revealed that T-DXd showed superior efficacy in terms of PFS (11.56 months) and ORR(53–57%). The safety profile of each treatment demonstrated distinct AE incidence profiles, though differing monitoring intensities across regimens may affect AE detection rates.

**Conclusions:**

Multiple endocrine-based strategies, including oral SERDs, AKT inhibitors combined with ET, and continued CDK4/6 inhibition, demonstrated clinical activity for patients progressing after CDK4/6 inhibitors. ADCs are superior to chemotherapy, with T-DXd showed the highest efficacy regarding PFS and ORR. This analysis provides a structured, evidence-based overview of the current therapeutic landscape for this clinically challenging population, with some limitations in directly comparing endocrine therapies and ADCs within the present analysis framework.

**Supplementary Information:**

The online version contains supplementary material available at 10.1186/s12885-026-15632-z.

## Introduction

Breast cancer (BC) is the most common malignancy among women worldwide, with approximately 2.3 million new cases diagnosed in 2022 [1]. Despite advances in screening and early diagnosis, approximately 30–40% of women with early-stage BC progress to advanced disease stages, when curative interventions are often not feasible, switching treatment goals toward prolonging survival and improving quality of life [2, 3]. Hormone receptor-positive (HR+) and HER2-negative (HER2-) BC are the most prevalent subtypes, comprising approximately 70% of cases of metastatic or advanced disease [2, 4]. The introduction of cyclin-dependent kinase 4/6 inhibitors (CDK4/6i) has revolutionized the treatment landscape for HR+/HER2- advanced BC. CDK4/6i have been confirmed as first-line treatments, with palbociclib approved in 2015, followed by ribociclib and abemaciclib in subsequent years [5–7].

Despite CDK4/6i revolutionizing treatment, resistance inevitably develops, necessitating optimized post-progression strategies [8, 9]. Following disease progression on CDK4/6i, treatment decisions become more complex due to the availability of various therapies but limited data to guide optimal strategy. Subsequent therapeutic options include ET monotherapy or combinations such as ET combined with everolimus or another CDK4/6i. For patients harboring PI3K mutations, treatment options include ET combined with alpelisib or capivasertib. In cases of ESR1 mutations, oral selective estrogen receptor degraders (SERDs) are recommended as part of the therapy [10]. For patients with HER2-low- or HER2-ultralow-expressing tumors, trastuzumab deruxtecan (T-DXd), an anti-HER2 antibody–drug conjugate (ADC), has demonstrated robust clinical efficacy. Additionally, Trop2-targeted ADCs, such as sacituzumab govitecan (SG) and datopotamab deruxtecan (Dato-DXd), have become significant therapeutic options for this population [10].

Recent guidelines suggest a nuanced approach that tailors treatment based on individual patient factors, such as prior treatment responses, mutational status, and overall health [11, 12]. However, comparative data on the efficacy and safety of available treatments in HR + and HER2- populations previously treated with CDK4/6i is limited, hindering the establishment of evidence-based sequencing strategies. The emergence of new therapies further complicates treatment decisions. To address this gap, this systematic review and network meta-analysis (NMA) comprehensively evaluates and compares the therapeutic efficacy and safety profiles of diverse treatment regimens following CDK4/6i therapy. It provides a rigorous, evidence-based synthesis of the current therapeutic landscape, elucidates existing knowledge gaps, and offers robust clinical evidence to inform treatment decisions for HR + and HER2- advanced BC patients with disease progression after CDK4/6i treatment.

## Methods

This study followed the PRISMA 2015 NMA Checklist [13]. The protocol was registered on PROSPERO (registration number CRD420250610285).

### Data sources and literature search

We searched PubMed, Embase, and the Cochrane Library for eligible studies published between 2015 and 2024. We set 2015 as the starting time point because palbociclib, the first drug in the CDK4/6i class, was launched globally that year. There were no restrictions on languages or publication status. Search terms included the following keywords and their synonyms: “breast neoplasms,” “human epidermal growth factor receptor 2 negative,” “HER2-negative”, “HER2-low”, “HR positive,” “previously treated,” “pretreated,” and “randomized control trial.” Details of the search strategy are provided in Supplementary 1. To minimize the risk of missing important articles, we also contacted clinical experts to identify additional relevant studies.

### Study selection

Studies were included if they were (1) designed as randomized controlled trials (RCTs); (2) enrolled CDK4/6i-treated patients with HR + and HER2-(negative or low) inoperable, locally advanced, or metastatic BC, with ≥ 80% of the population having received CDK4/6i therapy or reporting data on a CDK4/6i-treated subgroup; or (3) evaluated the efficacy and safety of monotherapy or combined ET (including CDK4/6i combinations, targeted drugs combined with ET, SERDs etc.), ADCs (such as T-DXd, SG, and Dato-DXd), or chemotherapy. The primary outcome of interest was PFS, as defined in the original studies. The secondary outcome was objective response rate (ORR), as defined in original studies. Specifically, we collected data on overall grade 3 or higher AEs, as well as individual AEs with an incidence greater than 10%. Studies were excluded if they (1) had a sample size of fewer than 20 participants; (2) evaluated treatments that had failed in previous trials; (3) included fewer than 80% CDK4/6i-treated patients, or lacked a CDK4/6i-treated subgroup analysis. Two reviewers independently screened articles for eligibility. Any disagreements were resolved through discussion, with assistance from a third reviewer if necessary.

### Data extraction

Two reviewers extracted the following data from each study into a pre-defined data extraction form: general study characteristics (e.g., NCT number, study title/trial name, first author name, publication year), study design (e.g., RCT phase, parallel/crossover design, sample size, blinding, location, analysis population, follow-up time), participants’ baseline characteristics (e.g., number of previous treatment lines, prior treatments, endocrine resistance status, site of metastasis, country, diagnostic criteria, age, sex), treatment design (e.g., interventions, dosing regimens, route of administration, treatment duration, length of follow-up time, total study duration), outcomes (primary and secondary outcome indicators as mentioned above). Any disagreements were resolved by discussion, with assistance from a third reviewer if necessary.

### Data synthesis and analysis

A Bayesian fixed-effects model was employed assuming a common true treatment effect across studies for each outcome. Log odds ratios (ORs) and hazard ratios (HRs) were estimated directly across the network, with treatment effects modeled as fixed parameters. The network structure incorporated direct comparisons, and indirect evidence was synthesized under the consistency assumption. Markov chain Monte Carlo sampling was conducted using JAGS or Stan with 50,000 iterations, including a burn-in of 20,000 iterations. Convergence was assessed through trace plots and Gelman-Rubin diagnostics. Results were summarized as posterior medians with 95% credible intervals (CIs), and treatment rankings were derived from posterior probabilities.

### Prior Assumptions

- Treatment Effects: Non-informative priors were assigned to all treatment effect parameters to minimize prior influence.
- Consistency: Consistency equations were enforced to ensure coherence between direct and indirect evidence, assuming no systematic differences in effect modifiers across comparisons.

### Implementation

Markov Chain Monte Carlo (MCMC) sampling was conducted using JAGS or Stan, with 50,000 iterations, including a burn-in of 20,000. Convergence was assessed through trace plots and Gelman-Rubin diagnostics. Results were summarized as posterior medians with 95% credible intervals (CIs), and treatment rankings were derived from posterior probabilities. Effect sizes were reported as HRs with 95% CIs for PFS and as ORs with 95% CIs for ORR. League tables were generated for direct comparisons of treatment effects. Treatment ranking probabilities were computed using the surface under the cumulative ranking curve (SUCRA), with higher values indicating a greater likelihood of being the optimal choice [14]. Since no closed loops existed in the network (i.e., no multiple indirect comparison pathways existed between treatments), a consistency model was applied without the need for node-splitting analysis [15]. Between-study heterogeneity was evaluated using the I² statistic, with values ≥ 50% indicating substantial heterogeneity [16].

To estimate the absolute effect of each treatment on PFS and ORR, we employed descriptive statistical methods to pool the results of individual studies. For PFS, survival curves for each intervention were pooled using the metaSurvival and IPMforsurv packages in R. Additionally, a bubble plot was generated to visualize ORR, where bubble size represented sample size, colors indicated different median follow-up times, and numbers in bubbles denoted ORR. Adverse events (AEs) from included studies were summarized in tables.

### Quality assessment

Two independent reviewers assessed the quality of RCTs using the Cochrane risk-of-bias tool [17]. Disagreements were resolved by discussion, with assistance from a third reviewer if necessary.

## Results

### Selection and characteristics of included studies

**Fig. 1:**
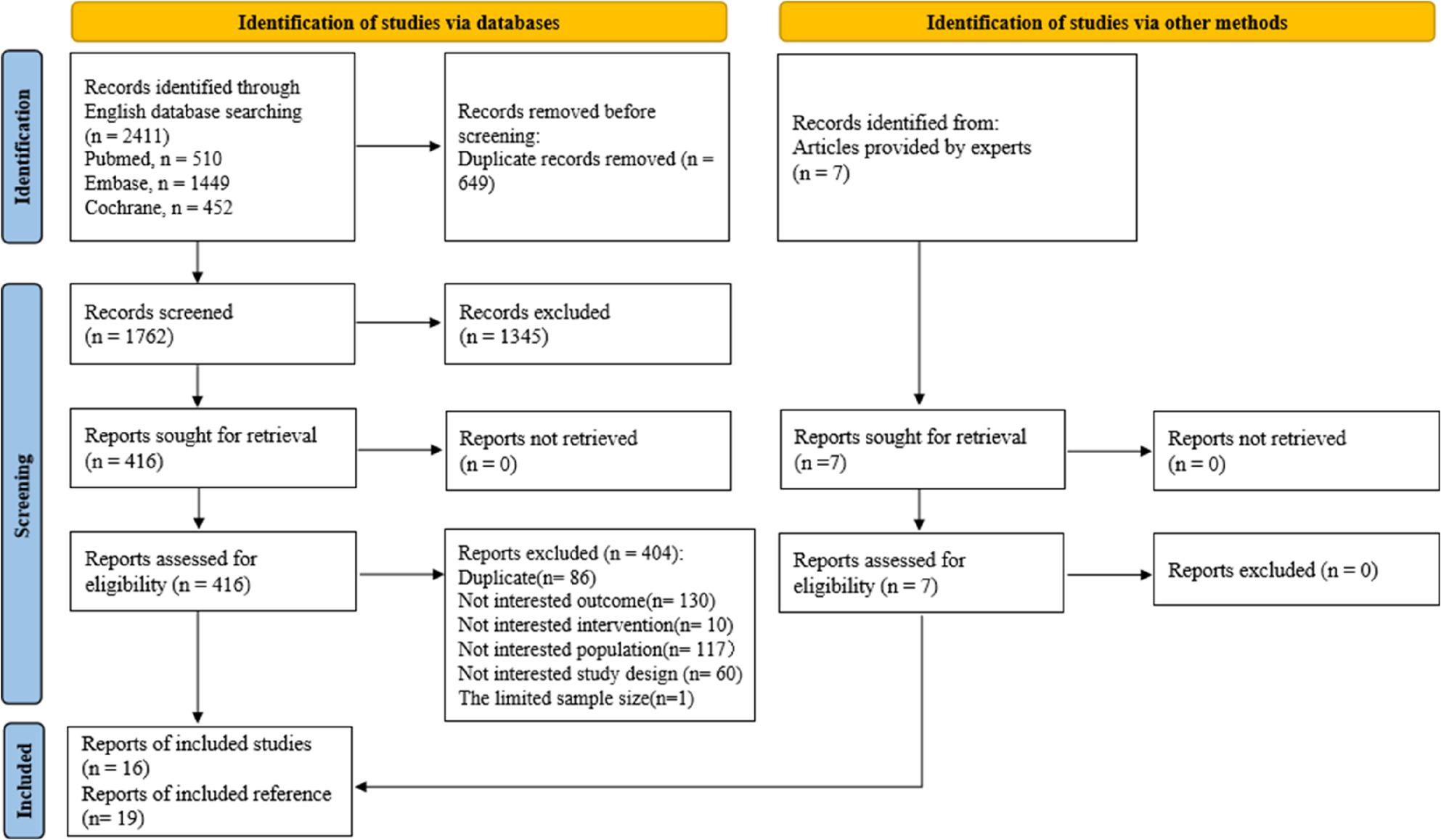
Flowchart illustrating the selection of studies

To avoid missing relevant studies, we first searched for RCTs investigating current treatments for HR+/HER2- advanced BC patients without restrictions on prior CDK4/6i treatment, identifying 1,762 unique references. We then manually screened eligible studies and included RCTs that assessed current treatments for HR+/HER2- advanced BC patients treated with CDK4/6i. After screening titles and abstracts, we excluded 1,346 references due to ineligible populations, treatments, or study designs, leaving 416 full texts for further review. In the full-text screening, we selected studies in which the entire population had received CDK4/6i therapy. For studies with a mixed population (i.e., not all patients received CDK4/6i therapy), we included those where at least 80% of patients had been treated with CDK4/6i or where outcome data were reported for the CDK4/6i-treated subgroup. Ultimately, 12 references were included from the electronic study search process. Moreover, manual searches of reference lists and expert consultation identified seven more eligible studies. In total, 16 RCTs (19 references, *n* = 5,076), published between 2015 and 2024, were included in the NMA. In ten studies [18–27], over 80% of patients had received prior CDK4/6i therapy, while in six other studies [28–33], the proportion of the population treated with CDK4/6i was less than 80% with data had been reported for the eligible subgroup. A detailed flowchart of the selection process, aligned with Preferred Reporting Items for Systematic Reviews and Meta-Analyses (PRISMA) guidelines, is provided in Fig. 1.

The characteristics of included studies were presented in Table 1. All 16 RCTs were multicenter studies. All study populations were CDK4/6i-treated HR+/HER2- advanced BC patients, however the prior lines of therapies vary across studies. Eight studies [18, 23, 24, 28, 29, 31–33] did not report prior lines of therapy, while the other eight studies [19–22, 25–27, 30] documented the treatment history. One study [21] included patients with a history of one prior treatment, two studies [19, 27] included patients with two prior treatments, and the remaining five studies [20, 22, 25, 26, 30] encompassed mixed populations with treatment histories ranging from one to four prior lines. Three studies [19, 22, 25] included patients who had received both ET and chemotherapy, four studies [20, 26, 27, 30] included those treated with ET only, and one study [21] did not specify prior treatments. Four studies compared CDK4/6i (ribociclib [22], abemaciclib [23], or palbociclib [24, 25]) combined with ET versus ET alone. Two studies investigated targeted therapies (capivasertib [31] or entinostat [33]) combined with ET versus ET alone. Five studies examined oral SERDs (elacestrant [19], lasofoxifene [21], amcenestrant [27], giredestrant [28], and camizestrant [30]) versus ET alone. These eleven studies contributed data for the network meta-analysis based on ET treatment. Five studies compared the ADCs Dato-DXd [18], T-DXd [20, 29], and SG [26, 32] versus chemotherapy, which contributed data for the network meta-analysis based on chemotherapy.

**Table 1:** Characteristics of included studies

Study ID	Trial name	Country	Intervention treatment	Comparison treatment	Age (i/c)	Sample size (i/c)	The proportion of CDK4/6i treated	No. of previous treatment lines (i/c)	No. of previous ET lines (i/c)	No. of previous chemotherapy lines (i/c)	Endocrine resistance status	Mutation status	Site of metastasis (i/c)
Aditya 2024	TROPION-Breast01 trial	Global	Datopotamab deruxtecan	Chemotherapy	56(29–86)/ 54(28–86)	365/367	79.92%	NR	NR	line 1: 229/225 line 2: 135/141	NR	NR	NR
Bidard 2022 [19]	EMERALD trial	Global	Elacestrant	ET	63(24–89)/ 64 (32–83)	239/238	100%	(ET + Chemo) line 1:129/141; line 2:110/97	line 1:129/141; line 2:110/97	i/c: line 0:191/180; line 1:48/58	NR	ESR1 mutation: 48.7%	Visceral metastasis:163/169
Goetz 2023 [21]	ELAINE 1 trial	United States, Canada, and Israel	Lasofoxifene	Fulvestrant	60.3 ± 11.6/ 61.2 ± 10.4	52/51	100%	(CDK4/6) line 1: 52/51;	NR	NR	NR	ESR1 mutation: 100%	Metastatic bone disease only:13/11; Visceral disease only:13/10; Both:18/23
Kevin 2023 [22]	MAINTAIN trial	United States	Ribociclib + ET	Placebo + ET	55(48–67)/59 (51.5–65)	60/59	100%	ET + Chemo): line 0:9/11; line 1:40/37; line 2:11/9༛ line 3:0/1༛ line 4:0/1	line 0: 9/11; line 1:40/37; line 2:11/9༛ line 3:0/1༛ line 4:0/1	line 0:56/52; line 1:4/7	NR	ESR1 mutation :42.3%; TP53 mutation: 30.3%; CCND1 alterations : 24.2%; PIK3CA mutations : 21.2%; FGFR1 alterations : 18.2%.	Visceral:36/35; bone only:13/9
Miguel 2024 [28]	acelERA Breast Cancer trial	Asia, Europe, Australia, North America, Africa, South America	Giredestrant	Fulvestrant or AI	60.0(28–85)/59.0(32–93)	152/151	43%	line 1: 103/113 line 2: 47/38	(Al) Last line: 102/94	Last line: 34/40	NR	ESR1 mutation: 38.8%	Visceral:104/103; Measurable:141/14; CNS involvement: 3/3; Bone-only:14/14
Mayer 2024 [25]	PACE trial	United States	Fulvestrant + Palbociclib	Fulvestrant	58(36–77)/55 (28–77)	111/55	100%	(ET + Chemo): line 1:5/3; line 2:83/42; line > 2:21/10	NR	NR	Endocrine-resistant :32/10; Endocrine-sensitive:78/45	ESR1 mutations: 54%; PIK3CA mutations : 35%; RB1 alteration : 11.5%.	Visceral : 29/70; Bone-only :4/18
Modi 2022 (Harbeck 2022) [29]	DESTINY-Breast04 trial	Europe or Israel, Asia, North America	Trastuzumab Deruxtecan	Physician’s Choice of Chemotherapy	56.8(31.5–80.2)/55.7(28.4–80.0)	331/163	70.40%	line 1:23/14; line 2:85/41; line ≥ 3: 223/108	NR	NR	NR	NR	Brain:18/7; Liver:247/116 Lung:98/58
Oliveira 2024 [30]	SERENA-2 trial	Asia, Europe, the Middle East, and North America	Camizestrant	Fulvestrant	61(55–68)/60(55–70)	74/73	51%	NR	line 0:1/0 line 1:60/56 line 2:12/14 line 3:1/1	NR	NR	ESR1 mutations : 38.8%.	Liver or Lung:43/43 lung:23/35
Rugo 2022 (Rugo 2023) [26]	TROPiCS-02 trial	Global	Sacituzumab govitecan	Chemotherapy	57(29–86)/55 (27–78)	272/271	100%	(CDK4/6i): line 1:84/101; line 2:57/56; line 3:55/49; line 4 or more: 71/62;	NR	line 0: 1/0; line 1: 8/2; line 2: 104/118; line 3 or 4: 159/151	Endocrine resistant: 272/271	NR	Visceral :259/258; Liver :229/237
Tolaney 2023 [27]	AMEERA-3 trial	Global	Amcenestrant	ET	58(29–84)/60(28–86)	143/147	79%	NR	line 0: 9/10; line 1: 117/121; line 2: 17/16	NR	Primary resistance:8/6 Secondary resistance:134/141	ESR1 mutations : 41.4%.	Visceral:91/94; Bone-only :9/12
Turner 2023 [31] (Oliveira 2023)	CAPItello-291 trial	Australia, Canada, Israel, United States, Western Europe, Eastern Europe, Latin America, or Russia, Asia	Capivasertib + Fulvestrant	Fulvestrant + Placebo	59(26–84)/ 58(26–90)	355/354	69.10%	line 0: 37/52; line 1:235/208; line 2:73/77; line 3:10/16;	line 0: 39/54; line 1: 287/252; line 2: 29/47;	NR	Primary resistance:127/135 Secondary resistance:228/218	AKT pathway-altered: 40.8%.	Bone-only :51/52; Liver: 156/150; Viscera: 237/241
Xu 2023(a) [32]		China	Exemestane + Entinostat	Exemestane + Placebo	53.2 ± 10.71/51.9 ± 10.38	235/119	7%	line 0: 53/32; line 1:83/38; line 2:63/35; line 3:30/9; line 4: 6/5	NR	NR	Primary resistance:76/37	NR	Bone-only:32/23; Visceral disease: 162/82
Curigliano 2024 [20]	DESTINY-Breast06 trial	NR	Trastuzumab deruxtecan	Physician’s choice of chemotherapy	58(28–87)/ 57(32–83)	436/430	90.00%	line1:65/82; line2:295/288; line3+:75/58	line1:65/82; line2:295/288; line3+:75/58	NR	Primary resistance: 128/140;	NR	Bone-only:13/13; Visceral disease:376/364 Liver:296/283
Kevin 2024 [23]	postMONARCH trial	Global	Abemaciclib + Fulvestrant	Placebo + Fulvestrant	56(27–86)/ 61(28–85)	182/186	99%	NR	NR	NR	NR	ESR1 mutation : 45.3%; PIK3CA or AKT1 or PTEN mutation: 48.8%.	Bone only:18/23; Liver:37/38; Viscera:62/59
Llombart-Cussac 2023 [24]	PALMIRA trial	Global	Palbociclib + ET(letrozole or fulvestrant)	letrozole or Fulvestrant	59(33–85)/ 61(34–83)	136/62	100%	NR	NR	NR	NR	NR	Viscera:84/37
Xu 2023(b) [33]	EVER-132-002 trial	Asian(China, Taiwan, Korea)	Sacituzumab govitecan	Physician’s choice of chemotherapy	i/c: ≥ 65y: 15/23; < 65y:151/142	166/165	49%	NR	NR	line 2: 93/93; line 3–4: 73/72	NR	NR	Viscera:146/147

*AI* Aromatase inhibitors,* AKT1* serine/threonine kinase 1, *AKT* Protein kinase B, *c* comparison treatment group, *CCND1* Cyclin D1,* CDK4/6i* Cyclin-dependent kinase (CDK) 4/6 inhibition,* CNS* Central nervous system, *Chemo* Chemotherapy, *ET* Endocrine Therapy,* ESR1* Estrogen Receptor 1,* FGFR1* Fibroblast growth factor receptor 1, *i* intervention treatment group, *NR* Not reported, *PIK3CA* Phosphatidylinositol − 4,5 - bisphosphate 3 - kinase catalytic subunit alpha, *PTEN* Phosphatase and tensin homolog, *RB1 *Retinoblastoma protein 1, *TP53*,Tumor protein P53, *y* year

### Quality assessment of included studies

The overall risk of bias across the included studies was mixed (Supplementary Fig. 1). Random sequence generation was not adequately described, while allocation concealment was unclear in the majority of studies [18–20, 23–27, 29–33] (81%). Blinding of participants and personnel was reported in only five studies [20, 22, 23, 31, 33] (31%), while blinding of outcome assessment was adequately addressed in four studies [18, 19, 22, 27] (25%). Most studies had a low risk of incomplete outcome data and selective reporting, and no other potential sources of bias were identified. Most high-risk biases were associated with the blinding of participants, personnel, and outcome assessors. Nevertheless, since our outcome measures were objectively assessed, these biases are considered to have minimal impact on the overall findings.

### PFS

**Fig. 2:**
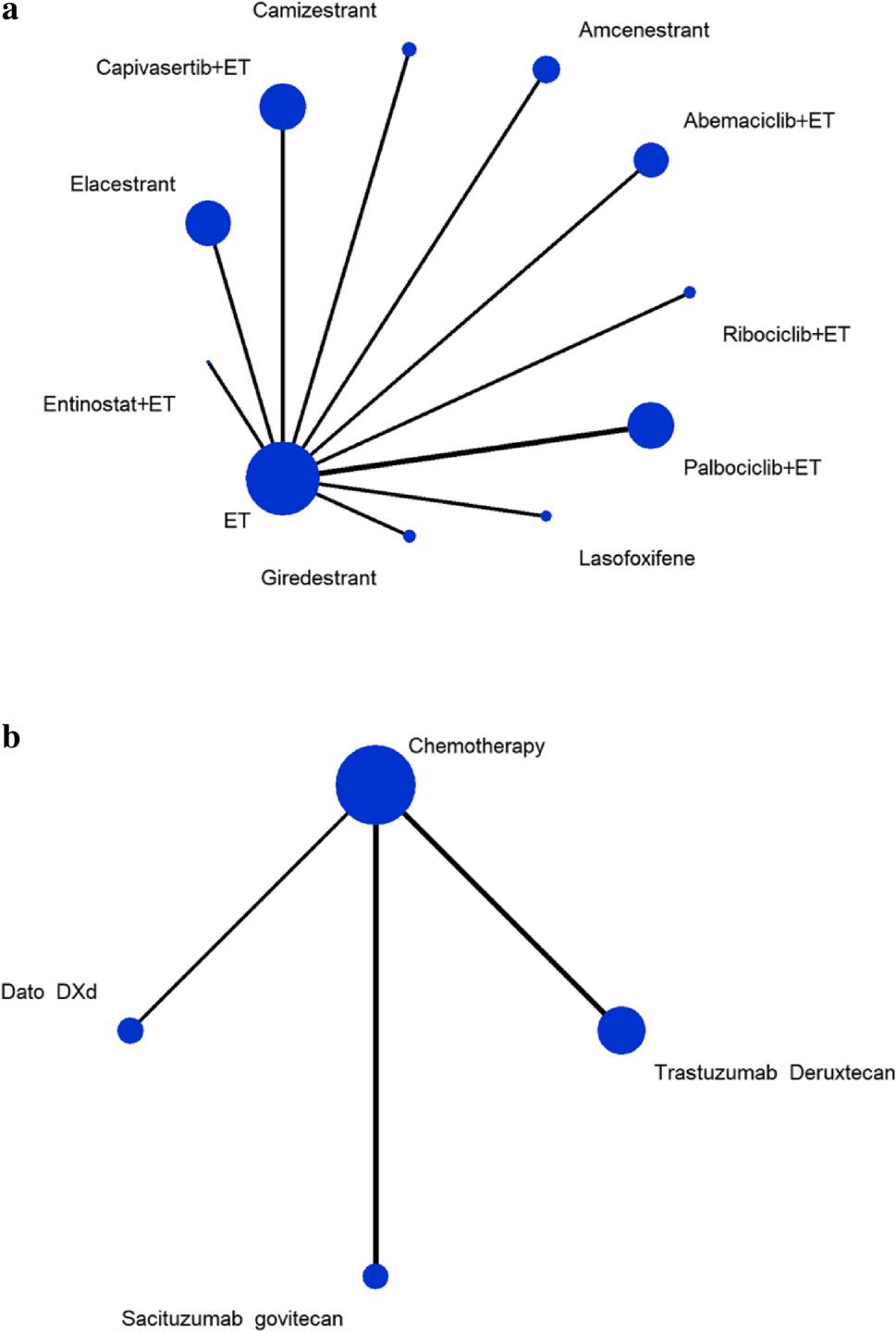
**a** Network plot of PFS based on ET treatment. **b** Network plot of PFS based on chemotherapy treatment

PFS indicators were analyzed in 16 studies [34]: ten studies [18–27] had a population where at least 80% had received CDK4/6i treatment, while six studies [28–33] reported data on a CDK4/6i-treated subgroup. No closed-loop were present in the network structure. Ultimately, two small networks were formed for analysis: one based on ET treatment and the other on chemotherapy (Fig. 2a and 2b). The ET treatment network included 11 studies [19, 21–25, 27, 28, 30, 31, 33] (*n* = 2,426) and 11 treatments: palbociclib plus ET, ribociclib plus ET, abemaciclib plus ET, capivasertib plus ET, entinostat plus ET, amcenestrant, camizestrant, elacestrant, giredestrant, lasofoxifene, and ET monotherapy. The chemotherapy network included five studies [18, 20, 26, 29, 32] (*n* = 2,650) and four treatments: Dato-DXd, SG, T-DXd, and chemotherapy.

The NMA of PFS in ET treatment network demonstrated that camizestrant (HR = 0.49, 95% CI: 0.32–0.76), ribociclib plus ET (HR = 0.57, 95% CI: 0.39–0.84), capivasertib plus ET (HR = 0.59, 95% CI: 0.48–0.72), elacestrant (HR = 0.70, 95% CI: 0.55–0.89), and abemaciclib plus ET (HR = 0.73, 95% CI: 0.57–0.94) significantly reduced the risk of disease progression or death compared with ET alone (Table 2). Other treatments, including entinostat plus ET, lasofoxifene, giredestrant, palbociclib plus ET, and amcenestrant, showed no significant difference from ET monotherapy (Table 2). Camizestrant (HR = 0.47, 95% CI: 0.27–0.82), ribociclib plus ET (HR = 0.55, 95% CI: 0.33–0.91), and capivasertib plus ET (0.57, 95% CI: 0.39–0.83) demonstrated a significantly reduced risk of disease progression or death compared with the amcenestrant group (Table 2). These treatments also showed a significant reduction in the risk of disease progression or death compared with palbociclib plus ET, with HRs (95% CIs) of 0.53 (0.33–0.86), 0.62 (0.40–0.95), and 0.64 (0.48–0.85), respectively (Table 2). The SUCRA values indicated that camizestrant ranked first in reducing the risk of disease progression or death, followed by ribociclib plus ET, capivasertib plus ET, entinostat plus ET, elacestrant, lasofoxifene, abemaciclib plus ET, giredestrant, palbociclib plus ET, amcenestrant, and ET monotherapy (Supplementary Table 1, Supplementary Fig. 2).

**Table 2:** League tables of PFS based on ET treatment

ET	0.73 (0.57, 0.94)	1.03 (0.75, 1.42)	0.49 (0.32, 0.76)	0.59 (0.48, 0.72)	0.7 (0.55, 0.89)	0.58 (0.22, 1.53)	0.8 (0.52, 1.23)	0.7 (0.43, 1.12)	0.92 (0.76, 1.12)	0.57 (0.39, 0.84)
1.37 (1.06, 1.77)	Abemaciclib plus ET	1.42 (0.94, 2.13)	0.67 (0.4, 1.12)	0.81 (0.58, 1.12)	0.96 (0.68, 1.36)	0.8 (0.29, 2.17)	1.1 (0.66, 1.81)	0.96 (0.56, 1.64)	1.26 (0.92, 1.75)	0.78 (0.49, 1.24)
0.97 (0.7, 1.33)	0.71 (0.47, 1.06)	Amcenestrant	0.47 (0.27, 0.82)	0.57 (0.39, 0.83)	0.68 (0.46, 1.01)	0.56 (0.2, 1.56)	0.77 (0.45, 1.32)	0.68 (0.38, 1.2)	0.89 (0.61, 1.3)	0.55 (0.33, 0.91)
2.04 (1.31, 3.17)	1.49 (0.89, 2.48)	2.11 (1.22, 3.64)	Camizestrant	1.2 (0.74, 1.96)	1.43 (0.87, 2.35)	1.19 (0.41, 3.44)	1.63 (0.88, 3.02)	1.43 (0.75, 2.72)	1.88 (1.16, 3.06)	1.16 (0.65, 2.09)
1.69 (1.38, 2.08)	1.24 (0.89, 1.71)	1.75 (1.2, 2.56)	0.83 (0.51, 1.35)	Capivasertib plus ET	1.19 (0.87, 1.62)	0.99 (0.37, 2.66)	1.36 (0.84, 2.18)	1.19 (0.71, 1.99)	1.57 (1.18, 2.08)	0.97 (0.62, 1.5)
1.43 (1.13, 1.81)	1.04 (0.74, 1.48)	1.48 (0.99, 2.19)	0.7 (0.42, 1.16)	0.84 (0.62, 1.15)	Elacestrant	0.83 (0.31, 2.26)	1.14 (0.7, 1.87)	1 (0.59, 1.7)	1.32 (0.97, 1.79)	0.81 (0.52, 1.28)
1.72 (0.65, 4.53)	1.26 (0.46, 3.41)	1.78 (0.64, 4.94)	0.84 (0.29, 2.45)	1.01 (0.38, 2.74)	1.2 (0.44, 3.27)	Entinostat plus ET	1.37 (0.48, 3.99)	1.2 (0.41, 3.55)	1.59 (0.59, 4.27)	0.98 (0.34, 2.79)
1.25 (0.81, 1.92)	0.91 (0.55, 1.5)	1.29 (0.76, 2.2)	0.61 (0.33, 1.13)	0.74 (0.46, 1.19)	0.87 (0.54, 1.43)	0.73 (0.25, 2.1)	Giredestrant	0.88 (0.46, 1.66)	1.15 (0.72, 1.85)	0.71 (0.4, 1.27)
1.43 (0.89, 2.3)	1.04 (0.61, 1.8)	1.48 (0.83, 2.63)	0.7 (0.37, 1.34)	0.84 (0.5, 1.42)	1 (0.59, 1.7)	0.83 (0.28, 2.45)	1.14 (0.6, 2.18)	Lasofoxifene	1.32 (0.79, 2.21)	0.81 (0.44, 1.51)
1.08 (0.89, 1.32)	0.79 (0.57, 1.09)	1.12 (0.77, 1.63)	0.53 (0.33, 0.86)	0.64 (0.48, 0.85)	0.76 (0.56, 1.03)	0.63 (0.23, 1.69)	0.87 (0.54, 1.39)	0.76 (0.45, 1.27)	Palbociclib plus ET	0.62 (0.4, 0.95)
1.75 (1.19, 2.59)	1.28 (0.81, 2.04)	1.81 (1.1, 3)	0.86 (0.48, 1.55)	1.04 (0.67, 1.61)	1.23 (0.78, 1.94)	1.02 (0.36, 2.9)	1.4 (0.79, 2.51)	1.23 (0.66, 2.27)	1.62 (1.05, 2.51)	Ribociclib plus ET

HR is the value for the drug in the column compared with the drug in the row

The NMA results for PFS indicators in the chemotherapy treatment (Table 3) showed that T-DXd, Dato-DXd, and SG were associated with a reduced risk of disease progression or death compared with chemotherapy, with HRs (95% CIs) of 0.59 (0.51–0.69), 0.63 (0.54–0.74), and 0.63 (0.52–0.76), respectively. Based on the SUCRA analysis, treatment regimens demonstrated hierarchical differences in disease progression or death risk, with T-DXd showing the lowest risk profile, followed sequentially by SG, then Dato-DXd, while conventional chemotherapy demonstrated the highest probability of disease progression or death (Supplementary Table 2).

**Table 3:** League tables of PFS based on chemotherapy treatment

Chemotherapy	0.63 (0.54, 0.74)	0.63 (0.52, 0.76)	0.59 (0.51, 0.69)
1.59 (1.36, 1.86)	Dato-DXd	1 (0.78, 1.27)	0.94 (0.75, 1.17)
1.59 (1.32, 1.92)	1 (0.78, 1.28)	SG	0.94 (0.74, 1.2)
1.69 (1.45, 1.98)	1.07 (0.85, 1.33)	1.06 (0.83, 1.36)	T-DXd

HR is the value for the drug in the column compared with the drug in the row

**Fig. 3:**
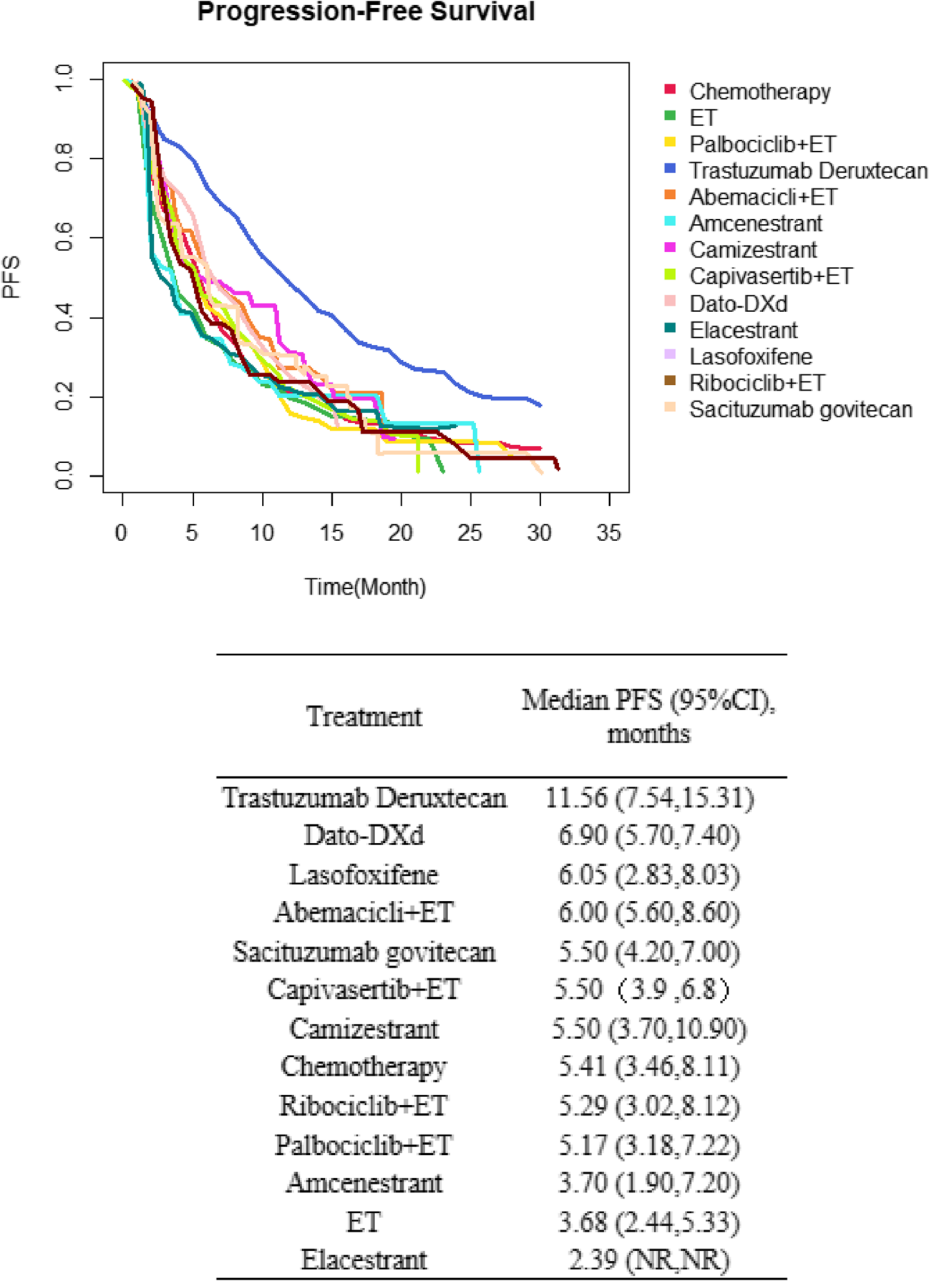
Synthesized Kaplan–Meier survival curves

Given the absence of a closed-loop network construction, PFS curves across treatment regimens were aggregated through meta-analysis to quantitatively assess the absolute treatment effects on PFS outcomes (Fig. 3). The combined survival curve (Fig. 3) indicated that T-DXd had the highest PFS probability (11.56 months; 95% CI: 7.54–15.31). Intermediate efficacy was observed with Dato-DXd (6.90 months; 95% CI: 5.70–7.40), lasofoxifene (6.05 months; 95% CI: 2.83–8.03), and combination therapies such as abemaciclib plus ET (6.00 months; 95% CI: 5.60–8.60). The lowest PFS values were reported for elacestrant (2.39 months; NR), amcenestrant (3.70 months; 95% CI: 1.90–7.20), and ET monotherapy (3.68 months; 95% CI: 2.44–5.33). Combinations like capivasertib plus ET (5.50 months; 95% CI: 3.90–6.80), ribociclib plus ET (5.29 months; 95% CI: 3.02–8.12) and palbociclib plus ET (5.17 months; 95%CI: 3.18–7.22) showed comparable efficacy to certain monotherapies (e.g., SG: 5.50 months, camizestrant: 5.50 months), but all were inferior to T-DXd. Conventional chemotherapy (5.41 months; 95% CI: 3.46–8.11) exhibited similar PFS to these therapies.

### ORR

**Fig. 4:**
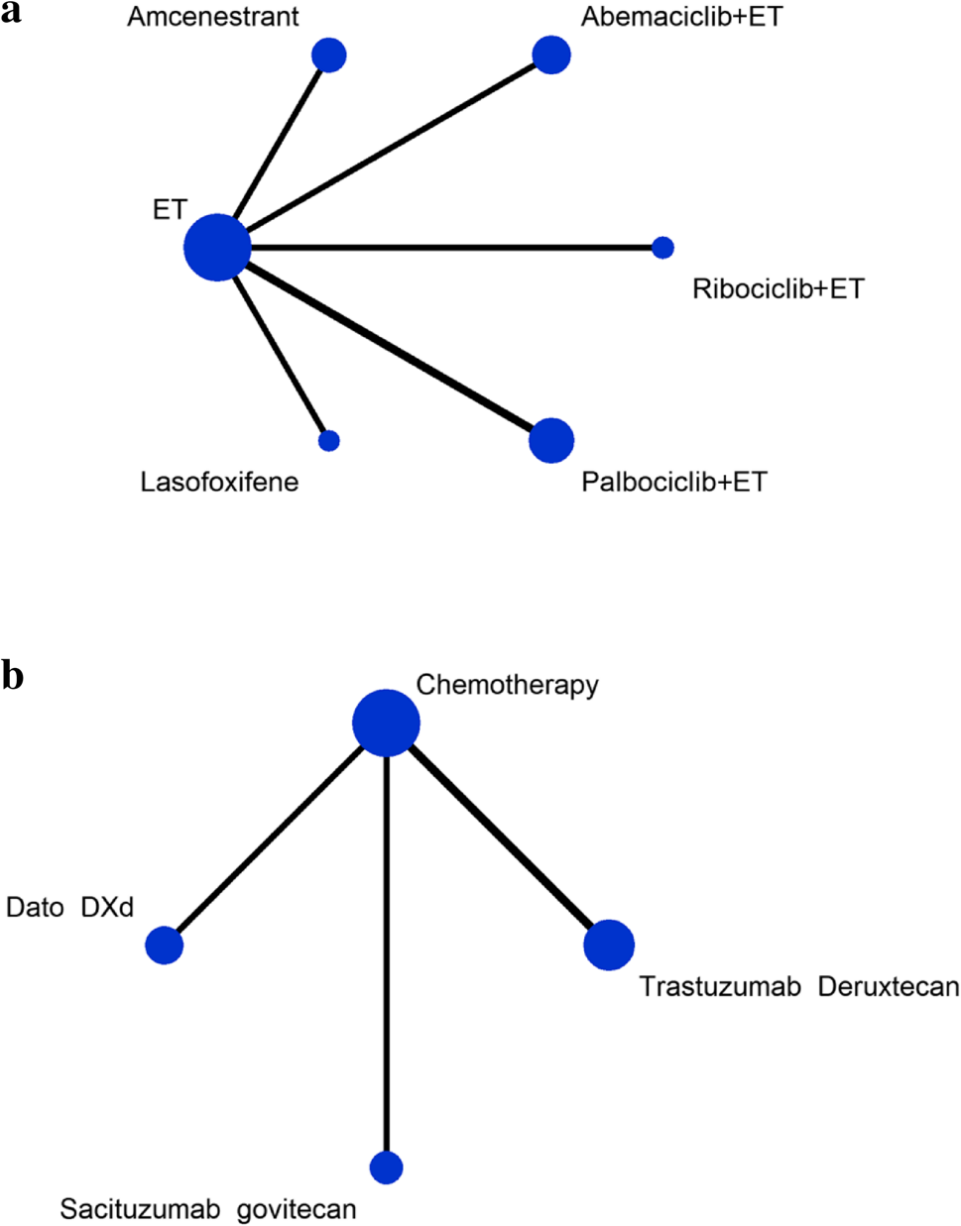
**a **Network plot of ORR based on ET treatment. **b **Network plot of ORR based on chemotherapy treatment

Among the 16 studies included in the analysis, three [19, 30, 33] failed to report ORR data, while three additional studies that enrolled heterogeneous populations did not provide subgroup-specific ORR data for patients receiving CDK4/6 inhibitor-based therapy. Consequently, data from 10 studies [18, 20–27, 29] were pooled in the meta-analysis, among which data from one study [29] were derived from its subgroup analysis. No closed-loop network was established, leading to the formation of two small networks based on ET treatment and chemotherapy (Fig. 4a and 4b). Six studies (*n* = 1,244) [21–25, 27] contributed data to the ET treatment network. The compared interventions included abemaciclib plus ET, amcenestrant, lasofoxifene, palbociclib plus ET, and ribociclib plus ET. Four studies (*n* = 2,489) [18, 20, 26, 29] contributed data for the chemotherapy network, with interventions including Dato-DXd, SG, T-DXd, and chemotherapy.

The NMA of ORR in ET treatment network (Table 4) showed that abemaciclib plus ET had a significantly higher ORR than ET alone (OR = 3.34, 95% CI: 1.48–8.19). The SUCRA analysis of ORR revealed that lasofoxifene demonstrating the highest efficacy, followed sequentially by abemaciclib plus ET, ribociclib plus ET, palbociclib plus ET, and amcenestrant, whereas ET monotherapy exhibited the lowest ORR (Supplementary Table 3, Supplementary Fig. 3).

**Table 4:** League tables of ORR based on ET treatment

ET	3.34 (1.48, 8.19)	1.4 (0.65, 3.05)	4.34 (0.91, 34.09)	1.65 (0.61, 5.25)	1.9 (0.7, 5.5)
0.3 (0.12, 0.68)	Abemaciclib plus ET	0.42 (0.13, 1.3)	1.3 (0.22, 11.92)	0.5 (0.13, 2.01)	0.57 (0.15, 2.18)
0.72 (0.33, 1.54)	2.39 (0.77, 7.74)	Amcenestrant	3.14 (0.54, 27.96)	1.18 (0.33, 4.65)	1.36 (0.38, 5)
0.23 (0.03, 1.1)	0.77 (0.08, 4.63)	0.32 (0.04, 1.86)	Lasofoxifene	0.38 (0.04, 2.65)	0.43 (0.04, 2.9)
0.61 (0.19, 1.63)	2.01 (0.5, 7.71)	0.85 (0.22, 2.99)	2.62 (0.38, 25.58)	Palbociclib plus ET	1.14 (0.25, 4.98)
0.53 (0.18, 1.43)	1.75 (0.46, 6.82)	0.74 (0.2, 2.63)	2.32 (0.35, 22.26)	0.88 (0.2, 4.05)	Ribociclib plus ET

OR is the value for the drug in the column compared with the drug in the row

In the chemotherapy NMA, ORR values for the T-DXd, Dato-DXd, and SG groups were significantly higher than for the chemotherapy group (Table 5), with ORs (95% CIs) of 3.53 (2.76–4.52), 1.94 (1.40–2.69), and 1.63 (1.04–2.58), respectively. ORR for the T-DXd group was also significantly higher than for the Dato-DXd group (OR = 1.82, 95% CI: 1.21–2.73) and the SG group (OR = 2.16, 95% CI: 1.29–3.62). The SUCRA ranking of ORR indicated that T-Dxd demonstrating the highest efficacy, followed sequentially by Dato-DXd, SG and chemotherapy.

**Table 5:** League tables of ORR based on chemotherapy treatment

Chemotherapy	1.94 (1.4, 2.69)	1.63 (1.04, 2.58)	3.53 (2.76, 4.52)
0.52 (0.37, 0.71)	Dato-DXd	0.84 (0.48, 1.47)	1.82 (1.21, 2.73)
0.61 (0.39, 0.96)	1.19 (0.68, 2.07)	SG	2.16 (1.29, 3.62)
0.28 (0.22, 0.36)	0.55 (0.37, 0.83)	0.46 (0.28, 0.78)	T-DXd

OR is the value for the drug in the column compared with the drug in the row

**Fig. 5:**
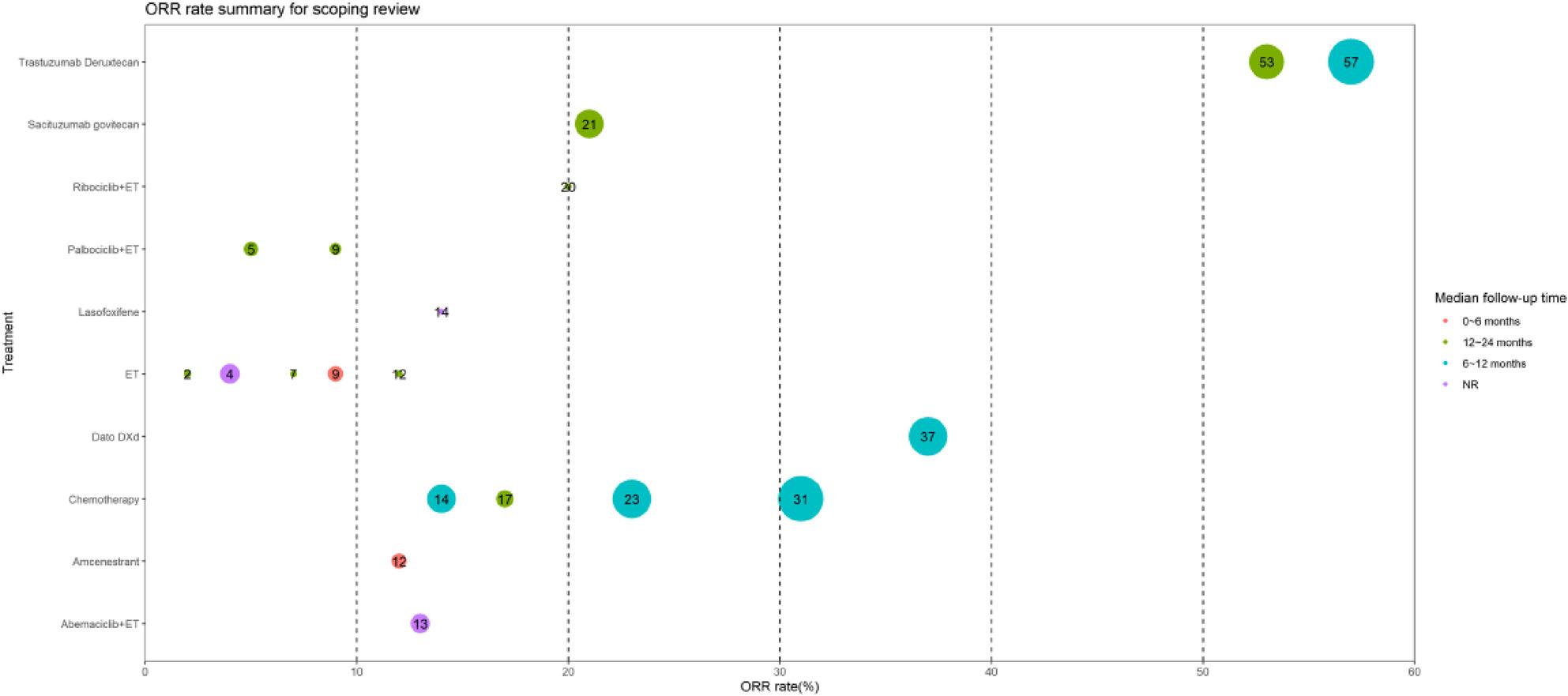
Bubble plot of ORR

Bubble plots (Fig. 5) quantitatively synthesized ORR values across regimens to evaluate absolute therapeutic efficacy. In general, ADCs and chemotherapy showed better effect on ORR: the ORR was significantly high for the T-DXd treatment group (53–57%), followed by Dato-DXd (37%), and chemotherapy (14%-31%) and SG (21%). The ORR of ribociclib plus ET were 20%, whereas other regimens – including palbociclib plus ET, lasofoxifene, ET monotherapy, amcenestrant, and abemaciclib plus ET– showed comparatively lower response (2–14%).

### AEs

Detailed AEs with an incidence rate above 10% were listed in Supplementary Table 4. In general, therapeutic agents, even within the same class, exhibit distinct AE incidence profiles. CDK4/6i (e.g., abemaciclib, palbociclib, ribociclib) are associated with high hematologic toxicity (neutropenia: up to 71.7% with ribociclib, 65.45% with palbociclib), as well as severe diarrhea (75% with abemaciclib) and drug-specific risks, such as QTc prolongation (13.3% treatment-emergent adverse events (TEAEs) with ribociclib), necessitating tailored monitoring (e.g., blood counts, ECG). Oral SERDs (e.g., amcenestrant, elacestrant) have a favorable safety profile, with low-to-moderate gastrointestinal toxicity (diarrhea: 10–14% TEAEs). Among the ADC drugs, T-DXd has unique risks of interstitial lung disease (ILD: 12.4%), SG shows high myelosuppression (70.5% neutropenia), and Dato-DXd has a low neutropenia rate (10.8%), suggesting intra-class safety variability.

Meanwhile, different regimens showed different incidence rate of severe AEs. CDK4/6i treatments exhibited significant toxicity, particularly for abemaciclib (grade ≥ 3 AEs: 55%, grade ≥ 3 neutropenia: 25%), palbociclib (grade ≥ 3 AEs: 45%, grade ≥ 3 neutropenia: 38.5%), and ribociclib (grade ≥ 3 neutropenia: 40%). Oral SERDs (e.g., amcenestrant, elacestrant, lasofoxifene) and ET monotherapy had the lowest AE burden (grade ≥ 3 AEs: 10–27%). ADCs showed marked variability: SG caused severe hematologic toxicity (grade ≥ 3 AEs: 73.9%, grade ≥ 3 neutropenia: 50.7%), while Dato-DXd had a milder profile (grade ≥ 3 AEs: 20.8%). T-DXd was associated with moderate toxicity (grade ≥ 3 AEs: 40.6–49.8%, grade ≥ 3 neutropenia: 20.7%) and frequent treatment disruptions (14.3% discontinuations). In comparison, chemotherapy had high grade ≥ 3 AE rates (44.7–66.1%), underscoring its role as a high-toxicity option.

## Discussion

Although CDK4/6 inhibitors have significantly improved the prognosis of patients with HR+/HER2- advanced breast cancer, drug resistance remains a major clinical challenge. In recent years, various novel therapies have emerged, but head-to-head comparisons are lacking. To address this gap, we conducted the NMA to compare the efficacy (PFS and ORR) and safety (AE patterns) of different treatment regimens in patients who experienced disease progression after CDK4/6i therapy, aiming to provide a comprehensive overview to guide clinical decision-making. This study demonstrated that patients with HR+/HER2- advanced breast cancer who progress following CDK4/6 inhibitor therapy have access to multiple novel therapeutic regimens exhibiting distinct efficacy and safety profiles. For efficacy outcomes, two treatment networks were constructed to compare regimens with endocrine therapy and chemotherapy, respectively.

In the ET-based network, several novel agents were compared with ET monotherapy, including oral SERD, AKT inhibitor, HDAC inhibitors. These novel agents with distinct mechanisms have been introduced into clinical practice in recent years. This NMA revealed that oral SERDs (camizestrant, elacestrant), AKT inhibitors (capivasertib) plus ET, and CDK4/6 inhibitors plus ET (abemaciclib, ribociclib) significantly outperformed ET monotherapy in PFS outcomes, which underscore the remained effective of the above pharmacologically distinct agents following CDK4/6 inhibitor progression. The SUCRA analysis suggested that camizestrant might be the most efficacious treatment. Additionally, comparisons of PFS effects among these agentswere conducted, providing evidence to aid clinical decision-making based on biomarker profiles and clinicopathological characteristics. Results showed that camizestrant, ribociclib plus ET, and capivasertib plus ET demonstrated superior PFS compared with amcenestrant or palbociclib plus ET. Regarding ORR, comparisons between agents were limited due to the absence of post-CDK4/6 subgroup reporting in some trials. Our analysis suggested that abemaciclib demonstrated a higher ORR among ET-based strategies. However, it should be noted that no single class of agents can be deemed universally superior across all clinical scenarios. Our results also highlight the potential of novel therapies to improve outcomes for patients among endocrine treatment based therapy, emphasizing the importance of personalized treatment approaches.

The observed differences in clinical efficacy among oral SERDs in the post-CDK4/6i setting may be partly explained by their distinct pharmacological properties. Camizestrant is a highly potent, next-generation oral SERD that achieves near-complete ER degradation (> 95%) at clinically relevant doses, exhibits high oral bioavailability, and effectively antagonises both wild-type and ESR1-mutated receptors due to its favourable binding kinetics and brain penetrance [35]. In contrast, elacestrant, while the first approved oral SERD, demonstrates lower ER degradation efficiency (∼70–90%) and has reduced potency against certain ESR1 mutations (e.g., Y537S), which may contribute to its more modest PFS benefit compared with camizestrant [35]. Amcenestrant and giredestrant failed to show significant improvement over standard endocrine therapy in phase II/III trials, likely due to insufficient receptor degradation, suboptimal pharmacokinetics, and/or inadequate target engagement in heavily pretreated patients. These pharmacological differences underscore the importance of selecting SERDs with optimal potency and exposure for ESR1-mutated tumours.

The differential clinical outcomes observed among AKT inhibitors in HR+/HER2- advanced breast cancer are largely attributable to differences in isoform selectivity, potency, and pharmacokinetic profiles. Capivasertib is an ATP-competitive pan-AKT inhibitor with balanced nanomolar potency against AKT1 (IC50 3 nmol/L), AKT2 (9 nmol/L), and AKT3 (3 nmol/L), enabling effective inhibition of downstream signalling even in the presence of compensatory isoform upregulation commonly seen after CDK4/6i resistance [31]. In contrast, earlier-generation pan-AKT inhibitors such as ipatasertib exhibit ∼10-fold lower potency against AKT2 and poorer CNS penetration, whereas isoform-selective agents (e.g., some AKT1-specific compounds) have failed in phase II/III trials due to incomplete pathway blockade and rapid emergence of AKT2/AKT3-mediated resistance [36]. Additionally, capivasertib demonstrates superior oral bioavailability and a longer half-life, supporting once-daily dosing and sustained target coverage in heavily pretreated patients [35]. These pharmacological advantages likely underlie the positive CAPItello-291 results and the negative outcomes of prior AKT inhibitors in similar settings.

In the chemotherapy-based network, ADCs showed significant benefit compared to chemotherapy alone, with T-DXd standing out as the most effective agent in both PFS and ORR. However, comparisons between ADCs and endocrine-based therapies are not feasible due to the separate networks. The superior efficacy of ADCs is largely attributed to its innovative mechanism in addressing the therapeuticwindow limitations of chemotherapy, offering more effective disease control with reduced side effects. Specifically, pooledd PFS curves showed that T-DXd was associated with the longest median PFS. Taken together, these results regarding PFS suggest that T-DXd may be a preferable choice among ADC options for patients with disease progression after CDK4/6i therapy, but direct comparisons with endocrine-based therapies are not possible due to separate network structures.

The superior efficacy of trastuzumab deruxtecan (T-DXd) over sacituzumab govitecan (SG) and datopotamab deruxtecan (Dato-DXd) in the post-CDK4/6i setting can be largely attributed to differences in target antigen expression, payload potency, linker stability, and bystander killing capacity. T-DXd employs a highly potent topoisomerase I inhibitor payload (DXd, IC50 ∼0.3 nmol/L) with a drug-to-antibody ratio (DAR) of ∼8 and a cleavable tetrapeptide linker that enables robust bystander effect across heterogeneous tumours, including those with low/heterogeneous HER2 expression. In contrast, SG uses a moderately potent topoisomerase I inhibitor (SN-38) with lower DAR (∼7.6) and a less stable pH-sensitive linker, resulting in greater systemic toxicity and limited bystander activity. Dato-DXd, despite its high-potency DXd payload, has a lower DAR (∼4) and more stable linker, which restricts bystander killing and may explain its intermediate efficacy in heavily pretreated HR + disease. These pharmacological distinctions, combined with higher HER2 expression relative to Trop-2 in many HR+/HER2-low tumours, likely drive the observed hierarchy of T-DXd > Dato-DXd ≈ SG.

The differential clinical outcomes observed among various ADCs in breast cancer can be attributed to differences in target antigen expression, payload potency and mechanism of action, linker stability, and bystander killing effects. For instance, trastuzumab deruxtecan (T-DXd) exhibits superior efficacy in HER2-low tumors due to its high-affinity HER2 targeting and potent, membrane-permeable DXd payload, which enables effective bystander killing of neighboring cells. In contrast, SG targets Trop-2 with broader expression but relies on SN-38, which has lower permeability and thus more limited bystander activity. Crucially, these pharmacological distinctions also dictate their divergent safety profiles. The specific toxicity of T-DXd, such as interstitial lung disease (ILD), is potentially linked to the target-independent uptake of the DXd payload in lung tissues, whereas the dose-limiting toxicities of SG, including neutropenia and diarrhea, are characteristic of the SN-38 payload and the hydrolyzable linker that may release the payload prematurely in circulation. These pharmacological factors, including optimized linker designs for efficient payload release, collectively contribute to T-DXd’s higher progression-free survival and objective response rates as well as the distinct adverse event spectrums compared to other ADCs [37, 38]. However, there remains a lack of studies directly comparing chemotherapy and endocrine therapy in the post-CDK4/6i setting. Although several studies have evaluated the choice between chemotherapy and endocrine including PEARL [34], Right Choice [35], BOLERO6 [36] and Young-PEARL [37], heterogeneous findings were observed. Some studies showed no significant difference between the two treatments, while others indicated that endocrine therapy may be more effective than chemotherapy. Notably, none specifically focused on the population with disease progression after CDK4/6i therapy. The recent introduction of novel agents, including oral SERDs, AKT inhibitors, and ADCs, has substantially improved treatment outcomes. Because of absence of head-to-head comparisons, our analysis is limited to a scoping review of PFS and ORR across the available therapies. In our findings, T-DXd demonstrated the longest median PFS among all evaluated regimens in the post-CDK4/6i setting, while ADCs collectively showed higher ORRs compared to endocrine-based therapies. This pattern aligns with their distinct mechanisms of action: ADCs deliver cytotoxic payloads directly to tumor cells, achieving both high efficacy and an optimized therapeutic window, whereas endocrine therapies primarily modulate hormone signaling to delay disease progression, with generally maintaining favorable safety profiles.

In terms of safety, treatment-related AEs varied across different therapeutic classes which suggesting that tailored regimen should be individualized based on patients’ specific clinical profiles. Furthermore, the more intensive monitoring typically associated with ADCs and chemotherapies compared to endocrine therapies may result in higher AE detection rates, indicating a potential ascertainment bias that should be considered when interpreting safety data. For example, proactive monitoring for myelosuppression and QTc interval prolongation is required duringCDK4/6is therapy [38, 39], whereas careful assessment of ILD risk is essential when using T-DXd [40, 41]. Furthermore, Oral SERDs and ET monotherapy were associated with the lowest AE burden, indicatinging their potential suitability for frail populations. When selecting anti-cancer regimens thorough evaluation, of AE risks-particularly grade ≥ 3 AEs, should precede treatment initiation, followed by continuous monitoring. For this purpose, the present study provides a comprehensive summary of the of AE incidence rates, offering clinically actionable insights. However, it is important to note that the reporting of AEs was heterogeneous across the included studies, which may be attributed to differences in AE definitions, reporting standards, follow-up durations, and patient populations. These design characteristics necessitate cautious interpretation of the safety findings. Therefore, the comparative safety profiles of the treatments should be considered with this limitation in mind.

The differential severity of AEs among CDK4/6 inhibitors can be scientifically explained by their distinct pharmacological properties, including kinase selectivity, pharmacokinetic profiles, and off-target effects. Ribociclib exhibits the highest risk of QTc prolongation due to its inhibitory effects on hERG potassium channels and CYP3A4-mediated drug interactions, leading to cardiac repolarization delays observed in 4.5–13.3% of patients. In contrast, abemaciclib is associated with greater gastrointestinal toxicity, such as severe diarrhea (up to 75% incidence), attributable to its broader CDK4/6 selectivity and higher potency in inhibiting intestinal epithelial cell proliferation. Palbociclib primarily causes hematological AEs, including profound neutropenia (up to 71.7%), resulting from its potent G1-phase cell cycle arrest in hematopoietic progenitor cells, without significant off-target cardiac or gastrointestinal effects. These differences underscore the importance of individualized CDK4/6 inhibitor selection based on patient comorbidities and monitoring strategies to mitigate AE severity [42, 43].

This systematic review has several strengths. Notably, it provides a comprehensive assessment of emerging therapies targeting CDK4/6i-treated populations, including Dato-DXd-a critical gap unaddressed in the previous study [44]. Furthermore, through network meta-analysis, we assessed the efficacy differences among available therapies in PFS and ORR, while complementary single-arm meta-analyses quantified absolute treatment effects of each treatment regimen. Critically, adherence to PRISMA standards ensures methodological rigor, enhancinging evidence validity and minimizing bias risks. However, this study also has some limitations. Insufficient reporting of patient-level characteristics prevented subgroup analyses stratified by prior therapy lines, Highlighting the need for future studies to evaluate treatment effects in these subgroups. Despite advances in precision oncology, the lack of available data prevented subgroup analyses based on biomarker-defined populations (e.g., ESR1 mutations, AKT pathway alterations, or HER2-low expression). This limitation restricts the strength of precision-oncology conclusions and should be considered when interpreting the results. We suggest more studies to assess treatment efficacyfor patients with different biomarkers in future. Furthermore, differences in treatment lines and the confounding effects from post-trial therapies limited the feasibility of an OS-focused analysis, prompting the present study mainly focus on PFS, ORR and adverse effects. Therefore, more prospective studies are warranted to assess the efficacy of these therapies on OS outcomes in the post-CDK4/6i setting.

OS data remain immature or unreported in the majority of trials included in this analysis, primarily due to short follow-up duration, extensive crossover, and high rates of subsequent therapies in this late-line setting. Consequently, a reliable network meta-analysis of OS could not be performed. In patients with advanced breast cancer, progression-free survival (PFS) and objective response rate (ORR) are widely accepted surrogate endpoints that strongly correlate with clinically meaningful outcomes, including delay of chemotherapy initiation and disease-related symptom control [42, 45]. The demonstration of overall survival (OS) benefit in this setting is often confounded by extensive post-progression treatments, making PFS and ORR the most robust and clinically actionable measures for evaluating therapeutic efficacy in post-CDK4/6 inhibitor populations [43].

An important clinical question in the post-CDK4/6i setting is how to select between oral SERDs and AKT inhibitors when continuing endocrine-based therapy. Current evidence indicates that the presence of actionable biomarkers is the primary driver of this decision. ESR1 mutations, detected in 30–40% of patients after progression on first-line aromatase inhibitor plus CDK4/6i, markedly reduce the efficacy of conventional endocrine therapy while remaining sensitive to more potent estrogen receptor antagonism or degradation. The phase III EMERALD trial demonstrated that elacestrant significantly improved PFS versus standard endocrine therapy exclusively in the ESR1-mutated subgroup (HR 0.55, 95% CI 0.39–0.77), with minimal benefit in ESR1-wildtype patients (HR 0.86, 95% CI 0.63–1.19) [19]. Similarly, exploratory analyses of camizestrant (SERENA-2 and SERENA-6) and giredestrant consistently showed the largest magnitude of benefit in ESR1-mutated tumours. In contrast, the AKT inhibitor capivasertib (CAPItello-291 trial) provided substantial PFS improvement regardless of ESR1 mutation status (HR 0.60 overall; HR 0.50 in PIK3CA/AKT1/PTEN-altered and HR 0.70 in non-altered tumours), but the benefit was most pronounced in patients harbouring PIK3CA, AKT1 or PTEN alterations (HR 0.50, 95% CI 0.38–0.65 versus HR 0.70, 95% CI 0.56–0.88 in unaltered tumours) [31]. Although PIK3CA, AKT1, and PTEN alterations all converge on the PI3K/AKT/mTOR signaling pathway, their positions within the cascade differ: PIK3CA mutations primarily drive upstream PI3K activation, while PTEN loss or AKT1 mutations lead to direct and sustained AKT kinase activity. Capivasertib, as a pan-AKT inhibitor, provides broad downstream pathway blockade, remaining effective even in the presence of upstream PI3K activation or compensatory mechanisms, whereas SERDs are particularly effective when ESR1 mutations drive resistance independently of PI3K pathway alterations. Importantly, in patients with co-existing ESR1 and PIK3CA/AKT1/PTEN alterations, capivasertib still retained robust activity (HR 0.52, 95% CI 0.36–0.76 in ESR1 co-mutated subgroup), whereas SERDs appear less effective when PI3K-pathway alterations dominate resistance mechanisms [31]. Beyond genomics, clinical factors also inform the choice: patients with aggressive visceral disease or primary endocrine resistance may derive greater early disease control from capivasertib, whereas those with indolent, bone-dominant disease and confirmed ESR1 mutation are ideal candidates for oral SERDs, which generally exhibit a more favourable tolerability profile (grade ≥ 3 adverse events 10–27% versus 50–60% with capivasertib plus fulvestrant). Therefore, routine next-generation sequencing of circulating tumour DNA or tissue at progression on CDK4/6i is strongly recommended to guide the preferential use of SERDs in ESR1-mutated, PI3K-pathway-wildtype tumours and AKT inhibition when PIK3CA/AKT1/PTEN alterations are present, irrespective of ESR1 status.Our network meta-analysis synthesized evidence from trials with inherent heterogeneity in patient characteristics, including prior lines of therapy, endocrine resistance patterns (e.g., primary vs. secondary resistance), biomarker distributions (e.g., ESR1 or PIK3CA mutations), and treatment histories. Such variability may introduce residual confounding and bias the indirect comparisons. For instance, studies with more heavily pretreated populations (e.g., ≥ 2 prior lines) might show attenuated treatment effects compared to those with fewer prior therapies. Similarly, differences in endocrine resistance status or biomarker prevalence could modulate the efficacy of targeted agents (e.g., capivasertib in AKT-altered tumors). Although we employed a fixed-effects model to mitigate between-study heterogeneity, the absence of patient-level data limited subgroup analyses to explore these effect modifiers. Consequently, the comparative estimates should be interpreted with caution, and future studies should prioritize stratified analyses to account for these factors.

Our quality assessment revealed that most included trials had insufficiently reported randomization and unclear allocation concealment, which could potentially introduce selection bias. Although the primary outcomes (PFS and ORR) are objective measures, such biases might affect the pooled estimates by inflating treatment effects. However, the consistency of results across studies and the use of Bayesian fixed-effects models may have mitigated some of these concerns. Future studies should improve reporting of randomization and allocation concealment to enhance the reliability of meta-analyses.

A major limitation of this network meta-analysis is the considerable clinical and methodological heterogeneity across the included trials, which may affect the reliability of indirect treatment comparisons. Key sources of heterogeneity include differences in the number of prior therapy lines (ranging from 1st-line post-CDK4/6i to ≥ 4 prior lines), patterns of endocrine resistance (primary vs. secondary), prevalence of actionable biomarkers (ESR1 mutation rates 0–100%; PIK3CA/AKT1/PTEN alteration rates 0–41%), visceral disease burden, and types of preceding CDK4/6 inhibitor. These factors are known effect modifiers: for example, heavily pretreated patients or those with primary endocrine resistance tend to derive smaller benefits from subsequent endocrine-based regimens, whereas biomarker-negative populations may underestimate the true effect of targeted agents such as capivasertib or oral SERDs. Although we applied a fixed-effects model and observed no closed loops (precluding formal inconsistency testing), residual confounding from these unbalanced characteristics cannot be ruled out and may bias the comparative hazard ratios and rankings. Consequently, while the overall direction of treatment effects is likely robust, the precise magnitude of differences between regimens should be interpreted cautiously, particularly when extrapolating to specific clinical subgroups. Future head-to-head trials with stratified designs or individual patient data network meta-analyses are needed to minimise this source of bias.

## Conclusion

Multiple endocrine-based strategies, including oral SERDs, AKT inhibitors combined with ET, and continued CDK4/6 inhibition, demonstrated clinical activity for patients progressing after CDK4/6 inhibitors. ADCs are superior to chemotherapy, with T-DXd showed the highest efficacy regarding PFS and ORR. This analysis provides a structured, evidence-based overview of the current therapeutic landscape for this clinically challenging population. Direct comparisons between endocrine therapies and ADCs were not feasible within the present network meta-analysis framework. 

## Supplementary Information

Supplementary Material 1.

## Data Availability

All data generated or analysed during this study are included in this published article and its supplementary information files.

## Abbreviations

ADCs: Antibody–drug conjugates
AEs: Adverse events
BC: Breast cancer
CDK4/6i: Cyclin-dependent kinase 4/6 inhibitor
CIs: Credible intervals
Dato-DXd: Datopotamab deruxtecan
ET: Endocrine therapy
HER2-: HER2-negative
HR+: Hormone receptor-positive
HRs: Hazard ratios
ORR: Objective response rate
ORs: Odds ratios
PFS: Progression-free survival
RCTs: Randomized controlled trials
SERDs: Selective estrogen receptor degraders
SG: Sacituzumab govitecan
SUCRA: Surface under the cumulative ranking curve
T-DXd: Trastuzumab deruxtecan
