# Supplementary material for: Efficacy and safety of treatments in HR+/HER2- advanced breast cancer after CDK4/6 inhibitor progression: a network meta-analysis and scoping review
Source: BMC Cancer. 2026 Feb 7;26:349. doi: 10.1186/s12885-026-15632-z (PMC12990551; doi:10.1186/s12885-026-15632-z)
Supplement: Supplementary file 1 — Supplementary Material 1. [file 12885_2026_15632_MOESM1_ESM.doc]

# Supplementary 1 Search strategy

2024-6-19

|  | Pubmed | Embase | Cochrane |
| --- | --- | --- | --- |
| Results | 510 | 1449 | 452 |
| Del-dup | 510 | 1112 | 140 |
| Total-RCT | 1762 | | |

Pubmed

1. "Breast Neoplasms"[Mesh] OR "Breast"[Mesh] OR breast[tw] OR mamma[tw] OR mammary[tw] OR lobular[tw]
2. "Neoplasm Metastasis"[Mesh] OR advanced[tw] OR metastat*[tw] OR "late stage"[tw] OR resistan*[tw] OR "stage III"[tw] OR "stage IV"[tw] OR "stage 3"[tw] OR "stage 4"[tw] OR "stage IIIC"[tw] OR "stage IIIB"[tw] OR unresect*[tw] OR "un-resect*"[tw] OR "ABC"[tw]
3. "human epidermal growth factor receptor 2 negative"[tw] OR "HER2-negative"[tw] OR "HER2-"[tw] OR "ERBB-2 receptor negative"[tw] OR "ERBB-2 negative"[tw] OR "HER 2-negative"[tw] OR "HER negative"[tw] OR "ErbB2 receptor negative"[tw] OR "ErbB2 negative"[tw] OR "Erb-B2 receptor negative"[tw] OR "Erb-B2 negative"[tw] OR "cerbB 2 negative"[tw] OR "Her2neu negative"[tw] OR "cerbB2 negative"[tw] OR "Her2-neu negative"[tw] OR "HER2-low"[tw]
4. "HR positive"[tw] OR "ER positive"[tw] OR "PR positive"[tw] OR "hormone receptor positive"[tw] OR "estrogen receptor positive"[tw] OR "Oestrogen receptor positive"[tw] OR "progesterone receptor positive"[tw] OR "progestrogen receptor positive"[tw]
5. #1 AND #2 AND #3 AND #4 3559
6. "second-line"[tw] OR "2nd-line"[tw] OR "2 line"[tw] OR "2L"[tw] OR "2-L"[tw] OR after[tiab] OR failure[tiab] OR fail[tiab] OR "first line progress*"[tw] OR "1st line progress*"[tw] OR "Recurrence"[Mesh] OR Relaps*[tw] OR Recurrenc*[tw] OR Recrudescenc*[tw] OR Refractor*[tw] OR "previously treated"[tw] OR pretreated[tw] OR pre-treated[tw] OR "re-treated"[tw] OR "third line"[tw] OR "3rd line"[tw] OR "3 line"[tw] OR "fourth line"[tw] OR "4th line"[tw] OR "4 line"[tw] OR "5th line"[tw] OR "5 line"[tw]
7. #5 AND #6 1171，二线、进展
8. "Review Literature as Topic"[Mesh] OR "Review" [Publication Type] OR Review[ti] OR "Case Reports" [Publication Type] OR "Case Report"[ti] OR "A Case"[ti]
9. #7 NOT #8 1385
10. ("controlled clinical trial"[pt] OR "Controlled Clinical Trials as Topic"[MeSH] OR "Random Allocation"[MeSH] OR "Double-Blind Method"[MeSH] OR "single-blind method"[MeSH] OR "Control Groups"[MeSH] OR "cross-over studies"[MeSH] OR random*[tiab] OR placebo[tiab] OR trial[tiab] OR groups[tiab] OR crossover[tiab] OR cross-over[tiab] OR singleblind*[tiab] OR double blind*[tiab] OR triple blind*[tiab] OR Factorial design*[tiab]) NOT ("Animals"[Mesh] NOT ("Humans"[Mesh] AND "Animals"[Mesh]))
11. #9 AND #10 AND ("2015/01/01"[Date - Publication] : "3000"[Date - Publication]) 510

EMBASE

1. 'breast cancer'/exp OR ((breast OR lobular OR mamma*) NEAR/3 (cancer* OR angiosarcoma* OR carcinogenesis* OR carcinoma* OR sarcoma* OR adenocarcinoma*)):ab,ti,kw
2. 'advanced cancer'/exp OR 'metastasis'/exp OR (advanced OR metastat* OR "late stage" OR resistan* OR "stage III" OR "stage IV" OR "stage 3" OR "stage 4" OR "stage IIIC" OR "stage IIIB" OR unresect* OR "un-resect*" OR "ABC"):ab,ti,kw
3. (("human epidermal growth factor receptor 2" OR "HER2" OR "ERBB-2 " OR "HER 2" OR "HER" OR "ErbB2" OR "Erb-B2" OR "cerbB 2" OR "cerbB2" OR "Her2neu") NEAR/3 negative):ab,ti,kw
4. ((HR OR ER OR PR OR "hormone receptor" OR estrogen OR Oestrogen OR progesterone OR progestrogen) NEAR/3 positive):ab,ti,kw
5. #1 AND #2 AND #3 AND #4 6608
6. 'recurrent disease'/exp OR 'second-line treatment'/exp OR 'fifth-line treatment'/exp OR 'fourth-line treatment'/exp OR 'third-line treatment'/exp OR 'relapse'/exp OR ("second-line" OR "2nd-line" OR "2 line" OR "2L" OR "2-L" OR "first line progress*" OR "1st line progress*" OR Relaps* OR Recurrenc* OR Recrudescenc* OR Refractor* OR "previously treated" OR pretreated OR "pre-treated" OR "re-treated" OR "third line" OR "3rd line" OR "3 line" OR "fourth line" OR "4th line" OR "4 line" OR "5th line" OR "5 line"):ab,ti,kw OR (after OR failure OR fail):ab,ti
7. #5 AND #6 3869
8. 'case report'/exp OR 'review'/exp OR [review]/lim OR (review OR "case report" OR "a case"):ti
9. #7 NOT #8 3275
10. ('controlled clinical trial'/exp OR 'Controlled Clinical Trial (Topic)'/exp OR 'double blind procedure'/de OR 'control group'/de OR 'crossover procedure'/de OR 'single blind procedure'/de OR 'triple blind procedure'/de OR 'placebo'/de OR 'randomization'/exp OR (random* OR trial OR groups OR placebo* OR crossover OR "cross-over" OR "Factorial design*" OR ((Doubl* OR Singl* OR tripl*) NEAR/2 Blind*)):ab,ti,kw) NOT (('nonhuman'/exp OR 'animal'/exp) NOT 'human'/exp)
11. #9 AND #10 AND [2015-2024]/py 1449

COCHRANE

#1 MeSH descriptor: [Breast Neoplasms] explode all trees 20230

#2 ((breast OR lobular OR mamma*)):ti,ab,kw 64317

#3 #1 OR #2 64317

#4 MeSH descriptor: [Neoplasm Metastasis] explode all trees 7550

#5 (advanced OR metastat* OR "late stage" OR resistan* OR "stage III" OR "stage IV" OR "stage 3" OR "stage 4" OR "stage IIIC" OR "stage IIIB" OR unresect* OR "un-resectable" OR "ABC"):ti,ab,kw 195816

#6 #4 OR #5 199072

#7 ((("human epidermal growth factor receptor 2" OR "HER2" OR "ERBB-2 " OR "HER 2" OR "HER" OR "ErbB2" OR "Erb-B2" OR "cerbB 2" OR "cerbB2" OR "Her2neu") NEAR/3 negative)):ti,ab,kw 3433

#8 (((HR OR ER OR PR OR "hormone receptor" OR estrogen OR Oestrogen OR progesterone OR progestrogen) NEAR/3 positive)):ti,ab,kw 5553

#9 #3 AND #6 AND #7 AND #8 1328

#10 ("second-line" OR "2nd-line" OR "2 line" OR "first line progress*" OR "1st line progress*" OR Relaps* OR Recurrenc* OR Recrudescenc* OR Refractor* OR "previously treated" OR pretreated OR "pre-treated" OR "re-treated" OR "third line" OR "3rd line" OR "3 line" OR "fourth line" OR "4th line" OR "4 line" OR "5th line" OR "5 line"):ti,ab,kw 130280

#11 #9 AND #10 518，2015-2024 452 trials

# Supplementary Figure 1 Risk of bias of studies


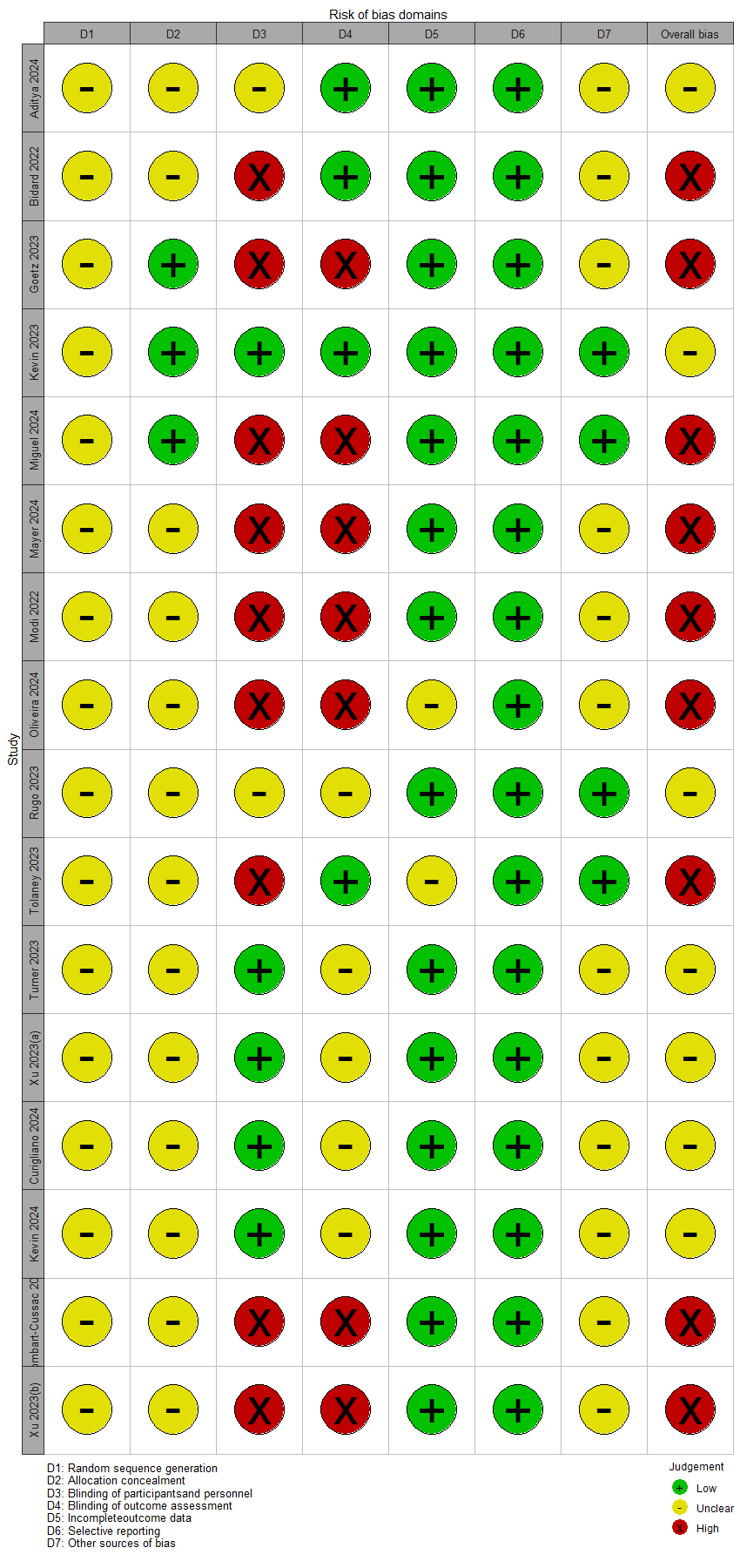


# Supplementary Table 1 SUCRA of PFS in ET treatment network

| **Treatment** | **SUCRA** |
| --- | --- |
| Camizestrant | 0.873 |
| Ribociclib plus ET | 0.764 |
| Capivasertib plus ET | 0.755 |
| Entinostat plus ET | 0.658 |
| Elacestrant | 0.549 |
| Lasofoxifene | 0.544 |
| Abemaciclib plus ET | 0.499 |
| Giredestrant | 0.396 |
| Palbociclib plus ET | 0.225 |
| Amcenestrant | 0.119 |
| ET | 0.116 |

# Supplementary Table 2 SUCRA of PFS in the chemotherapy treatment network

| **Treatment** | **SUCRA** |
| --- | --- |
| Trastuzumab Deruxtecan | 0.802 |
| Sacituzumab govitecan | 0.605 |
| Dato DXd | 0.594 |
| Chemotherapy | 0 |

# Supplementary Table 3 SUCRA of ORR in ET treatment network

| **Treatment** | **SUCRA** |
| --- | --- |
| Lasofoxifene | 0.821 |
| Abemaciclib plus ET | 0.791 |
| Ribociclib plus ET | 0.508 |
| Palbociclib plus ET | 0.44 |
| Amcenestrant | 0.339 |
| ET | 0.101 |

# Supplementary Table 4 AEs with an incidence rate above 10%

| **Study ID** | **Categories of Treatment** | **Treatment** | **Name of outcome** | **Events** | **Sample size** | **Incidence rate** | **NOTE** |
| --- | --- | --- | --- | --- | --- | --- | --- |
| Rugo 2023 | TROP2 ADC | Sacituzumab govitecan | Any TEAE ≥ grade3 | 198 | 268 | 73.88% | TEAE |
| Rugo 2023 |  |  | Neutropenia | 189 | 268 | 70.52% | TEAE |
| Rugo 2023 |  |  |  | 188 | 268 | 70.15% | TRAE |
| Rugo 2023 |  |  | Neutropenia ≥ grade3 | 136 | 268 | 50.75% | TRAE |
| Rugo 2023 |  |  | Leading to dose delay | 178 | 268 | 66.42% | TEAE |
| Rugo 2023 |  |  | Leading to dose reduction | 90 | 268 | 33.58% | TEAE |
| Rugo 2023 |  |  | Diarrhea | 166 | 268 | 61.94% | TEAE |
| Rugo 2023 |  |  |  | 152 | 268 | 56.72% | TRAE |
| Rugo 2023 |  |  | Nausea | 157 | 268 | 58.58% | TEAE |
| Rugo 2023 |  |  |  | 148 | 268 | 55.22% | TRAE |
| Rugo 2023 |  |  | Alopecia | 128 | 268 | 47.76% | TEAE |
| Rugo 2023 |  |  |  | 123 | 268 | 45.90% | TRAE |
| Rugo 2023 |  |  | Fatigue | 101 | 268 | 37.69% | TRAE |
| Rugo 2023 |  |  | Anemia | 98 | 268 | 36.57% | TEAE |
| Rugo 2023 |  |  |  | 91 | 268 | 33.96% | TRAE |
| Rugo 2023 |  |  | Serious TEAEs | 74 | 268 | 27.61% | TEAE |
| Rugo 2023 |  |  | Asthenia | 53 | 268 | 19.78% | TRAE |
| Rugo 2023 |  |  | Vomiting | 51 | 268 | 19.03% | TRAE |
| Rugo 2023 |  |  | Constipation | 50 | 268 | 18.66% | TRAE |
| Rugo 2023 |  |  | Decreased appetite | 42 | 268 | 15.67% | TRAE |
| Rugo 2023 |  |  | Leukopenia | 37 | 268 | 13.81% | TRAE |
| Rugo 2023 |  |  | Abdominal pain | 34 | 268 | 12.69% | TRAE |
| Rugo 2023 |  |  | Lymphopenia | 31 | 268 | 11.57% | TRAE |
| Aditya 2024 |  | Datopotamab deruxtecan | Any TRAE | 337 | 360 | 93.61% | TRAE |
| Aditya 2024 |  |  | Any TRAE ≥ grade3 | 75 | 360 | 20.83% | TRAE |
| Aditya 2024 |  |  | Associated with dose reduction | 75 | 360 | 20.83% | TRAE |
| Aditya 2024 |  |  | Associated with dose interruption | 43 | 360 | 11.94% | TRAE |
| Aditya 2024 |  |  | Nausea | 184 | 360 | 51.11% | TRAE |
| Aditya 2024 |  |  | Stomatitis | 180 | 360 | 50.00% | TRAE |
| Aditya 2024 |  |  | Alopecia | 131 | 360 | 36.39% | TRAE |
| Aditya 2024 |  |  | Fatigue | 85 | 360 | 23.61% | TRAE |
| Aditya 2024 |  |  | Dry eye | 78 | 360 | 21.67% | TRAE |
| Aditya 2024 |  |  | Vomiting | 71 | 360 | 19.72% | TRAE |
| Aditya 2024 |  |  | Constipation | 65 | 360 | 18.06% | TRAE |
| Aditya 2024 |  |  | Anemia | 40 | 360 | 11.11% | TRAE |
| Aditya 2024 |  |  | Neutropenia | 39 | 360 | 10.83% | TRAE |
| Harbeck 2022 | HER2 ADC | Trastuzumab Deruxtecan | Any TEAE | 221 | 223 | 99.10% | TEAE |
| Curigliano 2024 |  |  |  | 429 | 434 | 98.85% | TEAE |
| Curigliano 2024 |  |  | Any TRAE | 417 | 434 | 96.08% | TRAE |
| Harbeck 2022 |  |  | Any TEAE ≥ grade3 | 111 | 223 | 49.78% | TEAE |
| Curigliano 2024 |  |  | Any TRAE ≥ grade3 | 176 | 434 | 40.55% | TRAE |
| Curigliano 2024 |  |  | TEAEs associated with dose interruptions | 210 | 434 | 48.39% | TEAE |
| Curigliano 2024 |  |  | TEAEs associated with dose reductions | 107 | 434 | 24.65% | TEAE |
| Harbeck 2022 |  |  | Serious TEAEs | 58 | 223 | 26.20% | TEAE |
| Curigliano 2024 |  |  |  | 88 | 434 | 20.28% | TEAE |
| Harbeck 2022 |  |  | TEAEs leading to discontinuation | 33 | 223 | 14.60% | TEAE |
| Curigliano 2024 |  |  | TEAEs associated with treatment discontinuation | 62 | 434 | 14.29% | TEAE |
| Curigliano 2024 |  |  | Nausea | 286 | 434 | 65.90% | TRAE |
| Curigliano 2024 |  |  | Fatigue | 203 | 434 | 46.80% | TRAE |
| Curigliano 2024 |  |  | Alopecia | 197 | 434 | 45.40% | TRAE |
| Curigliano 2024 |  |  | Neutropenia | 163 | 434 | 37.60% | TRAE |
| Curigliano 2024 |  |  | Neutropenia ≥ grade3 | 90 | 434 | 20.70% | TRAE |
| Curigliano 2024 |  |  | Transaminases increased | 127 | 434 | 29.30% | TRAE |
| Curigliano 2024 |  |  | Anemia | 122 | 434 | 28.10% | TRAE |
| Curigliano 2024 |  |  | Vomiting | 118 | 434 | 27.20% | TRAE |
| Curigliano 2024 |  |  | Diarrhea | 103 | 434 | 23.70% | TRAE |
| Curigliano 2024 |  |  | Decreased appetite | 102 | 434 | 23.50% | TRAE |
| Curigliano 2024 |  |  | Leukopenia | 101 | 434 | 23.30% | TRAE |
| Harbeck 2022 |  |  | Interstitial lung disease | 28 | 223 | 12.40% | TRAE |
| Curigliano 2024 |  |  |  | 49 | 434 | 11.29% | TRAE |
| Kevin 2024 | CDK4/6i | Abemaciclib plus ET | Any TEAE | 176 | 181 | 97.00% | TEAE |
| Kevin 2024 |  |  | Any TEAE ≥ grade3 | 100 | 181 | 55.00% | TEAE |
| Kevin 2024 |  |  | Dose reductions due to AE | 55 | 181 | 30.39% | TEAE |
| Kevin 2024 |  |  | Diarrhea | 136 | 181 | 75.00% | TEAE |
| Kevin 2024 |  |  | Neutropenia | 74 | 181 | 41.00% | TEAE |
| Kevin 2024 |  |  | Neutropenia ≥ grade3 | 45 | 181 | 25.00% | TEAE |
| Kevin 2024 |  |  | Anemia | 63 | 181 | 35.00% | TEAE |
| Kevin 2024 |  |  | Fatigue | 60 | 181 | 33.00% | TEAE |
| Kevin 2024 |  |  | Nausea | 60 | 181 | 33.00% | TEAE |
| Kevin 2024 |  |  | Abdominal Pain | 43 | 181 | 24.00% | TEAE |
| Kevin 2024 |  |  | Vomiting | 36 | 181 | 20.00% | TEAE |
| Kevin 2024 |  |  | Decreased Appetite | 33 | 181 | 18.00% | TEAE |
| Kevin 2024 |  |  | Leukopenia | 33 | 181 | 18.00% | TEAE |
| Kevin 2024 |  |  | Thrombocytopenia | 33 | 181 | 18.00% | TEAE |
| Kevin 2024 |  |  | AST Increased | 27 | 181 | 15.00% | TEAE |
| Kevin 2024 |  |  | ALT Increased | 24 | 181 | 13.00% | TEAE |
| Kevin 2024 |  |  | Arthralgia | 22 | 181 | 12.00% | TEAE |
| Kevin 2024 |  |  | Cough | 20 | 181 | 11.00% | TEAE |
| Kevin 2024 |  |  | Creatinine Increased | 20 | 181 | 11.00% | TEAE |
| Llombart-Cussac 2023 |  | Palbociclib plus ET | Any TEAE | 125 | 135 | 92.59% | TEAE |
| Llombart-Cussac 2023 |  |  | Any TEAE ≥ grade3 | 62 | 135 | 45.93% | TEAE |
| Llombart-Cussac 2023 |  |  | Any TRAE | 99 | 135 | 73.30% | TEAE |
| Mayer 2024 |  |  | Any TRAE ≥ grade3 | 46 | 110 | 41.82% | TRAE |
| Llombart-Cussac 2023 |  |  |  | 51 | 135 | 37.80% | TRAE |
| Mayer 2024 |  |  | Palbociclib dose hold for toxicity | 40 | 110 | 36.36% |  |
| Mayer 2024 |  |  | Palbociclib dose reduction | 25 | 110 | 22.73% |  |
| Mayer 2024 |  |  | Neutropenia | 72 | 110 | 65.45% | TRAE |
| Llombart-Cussac 2023 |  |  |  | 71 | 135 | 52.59% | TEAE |
| Llombart-Cussac 2023 |  |  | Neutropenia ≥ grade3 | 52 | 135 | 38.52% | TEAE |
| Mayer 2024 |  |  |  | 36 | 110 | 32.73% | TRAE |
| Mayer 2024 |  |  | Fatigue | 38 | 110 | 34.55% | TRAE |
| Llombart-Cussac 2023 |  |  |  | 37 | 135 | 27.41% | TEAE |
| Mayer 2024 |  |  | Anemia | 24 | 110 | 21.82% | TRAE |
| Llombart-Cussac 2023 |  |  |  | 25 | 135 | 18.52% | TEAE |
| Llombart-Cussac 2023 |  |  | Arthralgia | 23 | 135 | 17.04% | TEAE |
| Mayer 2024 |  |  | Thrombocytopenia | 16 | 110 | 14.55% | TRAE |
| Llombart-Cussac 2023 |  |  | Nausea | 16 | 135 | 11.85% | TEAE |
| Mayer 2024 |  |  |  | 13 | 110 | 11.82% | TRAE |
| Mayer 2024 |  |  | Injection site reaction | 12 | 110 | 10.91% | TRAE |
| Llombart-Cussac 2023 |  |  | Leukopenia | 14 | 135 | 10.37% | TEAE |
| Mayer 2024 |  |  | Diarrhea | 11 | 110 | 10.00% | TRAE |
| Kevin 2023 |  | Ribociclib plus ET | Dose interruptions because of adverse events | 32 | 60 | 53.00% |  |
| Kevin 2023 |  |  | Dose reduction because of adverse events | 15 | 60 | 25.00% |  |
| Kevin 2023 |  |  | Neutropenia | 43 | 60 | 71.67% | TEAE |
| Kevin 2023 |  |  | Neutropenia ≥ grade3 | 24 | 60 | 40.00% | TEAE |
| Kevin 2023 |  |  | Fatigue | 20 | 60 | 33.33% | TEAE |
| Kevin 2023 |  |  | AST increased | 15 | 60 | 25.00% | TEAE |
| Kevin 2023 |  |  | Thrombocytopenia | 15 | 60 | 25.00% | TEAE |
| Kevin 2023 |  |  | Anemia | 14 | 60 | 23.33% | TEAE |
| Kevin 2023 |  |  | ALT increased | 10 | 60 | 16.67% | TEAE |
| Kevin 2023 |  |  | Diarrhea | 9 | 60 | 15.00% | TEAE |
| Kevin 2023 |  |  | QTc prolongation | 8 | 60 | 13.33% | TEAE |
| Kevin 2023 |  |  | Anorexia | 6 | 60 | 10.00% | TEAE |
| Kevin 2023 |  |  | Infection | 6 | 60 | 10.00% | TEAE |
| Tolaney 2023 | Oral SERD | Amcenestrant | Any TEAE | 118 | 143 | 82.52% | TEAE |
| Tolaney 2023 |  |  | Any TEAE ≥ grade3 | 31 | 143 | 21.68% | TEAE |
| Tolaney 2023 |  |  | Serious TEAEs | 23 | 143 | 16.08% | TEAE |
| Tolaney 2023 |  |  | Nausea | 29 | 143 | 20.28% | TEAE |
| Tolaney 2023 |  |  | Vomiting | 28 | 143 | 19.58% | TEAE |
| Tolaney 2023 |  |  | Arthralgia | 20 | 143 | 13.99% | TEAE |
| Tolaney 2023 |  |  | Back pain | 19 | 143 | 13.29% | TEAE |
| Tolaney 2023 |  |  | Headache | 18 | 143 | 12.59% | TEAE |
| Tolaney 2023 |  |  | Fatigue | 16 | 143 | 11.19% | TEAE |
| Tolaney 2023 |  |  | Diarrhea | 15 | 143 | 10.49% | TEAE |
| Bidard 2022 |  | Elacestrant | Any TEAE | 218 | 237 | 91.98% | TEAE |
| Bidard 2022 |  |  | Any TRAE | 150 | 237 | 63.29% | TRAE |
| Bidard 2022 |  |  | Any TEAE ≥ grade3 | 64 | 237 | 27.00% | TEAE |
| Bidard 2022 |  |  | Nausea | 83 | 237 | 35.02% | TEAE |
| Bidard 2022 |  |  |  | 60 | 237 | 25.32% | TRAE |
| Bidard 2022 |  |  | Fatigue | 45 | 237 | 18.99% | TEAE |
| Bidard 2022 |  |  |  | 26 | 237 | 10.97% | TRAE |
| Bidard 2022 |  |  | Vomiting | 45 | 237 | 18.99% | TEAE |
| Bidard 2022 |  |  |  | 26 | 237 | 10.97% | TRAE |
| Bidard 2022 |  |  | Decreased appetite | 35 | 237 | 14.77% | TEAE |
| Bidard 2022 |  |  | Arthralgia | 34 | 237 | 14.35% | TEAE |
| Bidard 2022 |  |  | Back pain | 33 | 237 | 13.92% | TEAE |
| Bidard 2022 |  |  | Diarrhea | 33 | 237 | 13.92% | TEAE |
| Bidard 2022 |  |  | AST increased | 31 | 237 | 13.08% | TEAE |
| Bidard 2022 |  |  | Constipation | 29 | 237 | 12.24% | TEAE |
| Bidard 2022 |  |  | Headache | 29 | 237 | 12.24% | TEAE |
| Bidard 2022 |  |  | Hot flush | 27 | 237 | 11.39% | TEAE |
| Bidard 2022 |  |  | Dyspepsia | 24 | 237 | 10.13% | TEAE |
| Goetz 2023 |  | Lasofoxifene | Any TEAE | 48 | 51 | 94.12% | TEAE |
| Goetz 2023 |  |  | Any TRAE | 33 | 51 | 64.71% | TRAE |
| Goetz 2023 |  |  | Any TEAE ≥ grade3 | 10 | 51 | 19.61% | TEAE |
| Goetz 2023 |  |  | Nausea | 14 | 51 | 27.45% | TEAE |
| Goetz 2023 |  |  | Fatigue | 12 | 51 | 23.53% | TEAE |
| Goetz 2023 |  |  | Arthralgia | 11 | 51 | 21.57% | TEAE |
| Goetz 2023 |  |  | Hot flush | 11 | 51 | 21.57% | TEAE |
| Goetz 2023 |  |  | Constipation | 8 | 51 | 15.69% | TEAE |
| Goetz 2023 |  |  | Cough | 8 | 51 | 15.69% | TEAE |
| Goetz 2023 |  |  | Dizziness | 8 | 51 | 15.69% | TEAE |
| Goetz 2023 |  |  | Hypertension | 8 | 51 | 15.69% | TEAE |
| Goetz 2023 |  |  | Anemia | 7 | 51 | 13.73% | TEAE |
| Goetz 2023 |  |  | Back pain | 7 | 51 | 13.73% | TEAE |
| Goetz 2023 |  |  | Diarrhea | 7 | 51 | 13.73% | TEAE |
| Goetz 2023 |  |  | Myalgia | 7 | 51 | 13.73% | TEAE |
| Goetz 2023 |  |  | Dyspnea | 6 | 51 | 11.76% | TEAE |
| Goetz 2023 |  |  | Muscle spasm | 6 | 51 | 11.76% | TEAE |
